# Allelic Interactions among *Pto-MIR475b* and Its Four Target Genes Potentially Affect Growth and Wood Properties in *Populus*

**DOI:** 10.3389/fpls.2017.01055

**Published:** 2017-06-21

**Authors:** Liang Xiao, Mingyang Quan, Qingzhang Du, Jinhui Chen, Jianbo Xie, Deqiang Zhang

**Affiliations:** ^1^Beijing Advanced Innovation Center for Tree Breeding by Molecular Design, College of Biological Sciences and Technology, Beijing Forestry UniversityBeijing, China; ^2^National Engineering Laboratory for Tree Breeding, College of Biological Sciences and Technology, Beijing Forestry UniversityBeijing, China; ^3^Key Laboratory of Genetics and Breeding in Forest Trees and Ornamental Plants, Ministry of Education, College of Biological Sciences and Technology, Beijing Forestry UniversityBeijing, China

**Keywords:** MiRNA, *Pto-MiR475b*, association study, allelic interaction, tree growth, wood formation

## Abstract

MicroRNAs (miRNAs) play crucial roles in plant growth and development, but few studies have illuminated the allelic interactions among miRNAs and their targets in perennial plants. Here, we combined analysis of expression patterns and single-nucleotide polymorphism (SNP)-based association studies to explore the interactions between *Pto-MIR475b* and its four target genes (*Pto-PPR1, Pto-PPR2, Pto-PPR3,* and *Pto-PPR4*) in 435 unrelated individuals of *Populus tomentosa*. Expression patterns showed a significant negative correlation (*r* = -0.447 to -0.411, *P* < 0.01) between *Pto-MIR475b* and its four targets in eight tissues of *P. tomentosa,* suggesting that Pto-miR475b may negatively regulate the four targets. Single SNP-based association studies identified 93 significant associations (*P* < 0.01, *Q* < 0.1) representing associations of 80 unique SNPs in *Pto-MIR475b* and its four targets with nine traits, revealing their potential roles in tree growth and wood formation. Moreover, one common SNP in the precursor region significantly altered the secondary structure of the pre-Pto-miR475b and changed the expression level of *Pto-MIR475b*. Analysis of epistatic interactions identified 115 significant SNP–SNP associations (*P* < 0.01) representing 45 unique SNPs from *Pto-MIR475b* and its four targets for 10 traits, revealing that genetic interactions between *Pto-MIR475b* and its targets influence quantitative traits of perennial plants. Our study provided a feasible strategy to study population genetics in forest trees and enhanced our understanding of miRNAs by dissecting the allelic interactions between this miRNA and its targets in *P. tomentosa*.

## Introduction

MicroRNAs (miRNAs) are small, non-coding RNAs of 20 to 24 nucleotides and play key roles in post-transcriptional regulation of gene expression in eukaryotes (He and Hannon, 2004; Rogers and Chen, 2013). MiRNAs function by cleaving the transcripts or suppressing the translation of their target genes through near-perfect sequence complementation (Chen, 2005). Plant miRNAs have diverse functions that are indispensable for development and growth (Jones-Rhoades et al., 2006). For example, At-miR166g and its target gene *At-HD-ZIP* co-regulate shoot apical meristem and lateral organ formation in *Arabidopsis thaliana* (Williams et al., 2005). Several recent studies have demonstrated that miRNAs are involved in various biological processes in trees, such as wood formation. For instance, Lu et al. (2013) overexpressed *Ptr-MIR397a* in nine transgenic *Populus trichocarpa* lines, which resulted in notably down-regulated expression of *Ptr-LACs* and decreased total laccase activity, which further reduced the Klason lignin content by as much as 22%. These results suggested that Ptr-miR397a is a master regulator of lignin polymerization and is involved in the regulation of the lignin biosynthesis pathway.

Trees provide essential ecosystem services and form an important part of the bioeconomy, as humans use trees for food, fuel, and biomaterials. However, the use of transgenic and reverse-genetic methods are difficult in forest trees due to their long generation times and high genome heterozygosity (Neale, 2007; Neale and Kremer, 2011). Using an alternative approach, recent candidate gene-based association studies have dissected the natural allelic variation responsible for specific traits in trees. This method has been successfully applied to identify major single-nucleotide polymorphisms (SNPs) associated with growth and wood formation in *Eucalyptus, Pinus*, and *Populus* (Dillon et al., 2010; Beaulieu et al., 2011; Du et al., 2015). For instance, Dillon et al. (2010) identified 10 SNPs from 38 candidate genes involved in the development of secondary xylem, which were significantly associated with solid-wood phenotypes in a natural population of *Pinus radiata.* Moreover, analysis of epistatic interactions revealed the mutual effects of gene–gene interactions on quantitative traits. Du et al. (2015) dissected the epistatic interactions of 11 key genes in the cellulose biosynthesis pathway using an epistasis model in *P. tomentosa*, which indicated that epistasis models can be used to investigate the interactions between different genes in the same pathway. In addition, association studies have been used to dissect the interactions between non-coding RNAs and genes. For instance, Quan et al. (2016) identified significant SNPs in *Pto-MIR156c* and *Pto-SPLs* (target genes of miR156c) by additive, dominant, and epistatic models, revealing the effect of their interactions on tree growth and wood formation in *P. tomentosa*.

With the development of next-generation sequencing, RNA-seq technology has been widely used to identify miRNAs that are differentially expressed spatiotemporally as well as in response to stress (Kozomara and Griffiths-Jones, 2011). For example, Xia et al. (2012) identified the conserved and specific miRNAs of five tissues in apple and Eldem et al. (2012) and Diler et al. (2016) found that differently expressed miRNAs respond to drought or hyperhydricity in peach. Therefore, illuminating the regulatory roles of miRNAs can improve our understanding of the mechanisms underlying the response to changing environments in perennial trees, and this information can be used in breeding programs for forest trees. In particular, miR475b was highly expressed in the developing xylem of *P. trichocarpa* (Lu et al., 2005). By comparing expression patterns in wild type and overexpressed the promoter of *MIR475b* lines in *Populus suaveolens*, Niu et al. (2016) found that miR475b responded to freezing stress and down-regulated pentatricopeptide repeat superfamily genes (*PPR*s). Although miR475b is highly expressed in the developing xylem, the allelic interactions of *MIR475b* and its targets have not been investigated, and the roles of miR475b in tree growth and wood formation are still unclear.

Here, we explored the allelic interactions of miR475b and its targets and their potential effects on tree growth and wood formation in *P. tomentosa*. We identified *Pto-PPR1, Pto-PPR2, Pto-PPR3,* and *Pto-PPR4* as putative targets of Pto-miR475b through bioinformatics prediction and degradome sequencing. We also analyzed their expression patterns to explore the correlation between *Pto-MIR475b* and its putative targets in eight tissues of *P. tomentosa*. Association studies in a population of 435 unrelated individuals of *P. tomentosa* allowed us to dissect the additive, dominant, and epistatic effects of *Pto-MIR475b* and the four putative targets to identify the complex regulatory network underlying tree growth and wood formation. In addition, we identified a common SNP in the pre-miRNA (precursor region) of *Pto-MIR475b* that altered the stem-loop structure of Pto-miR475b and changed the expression level of *Pto-MIR475b*. Our study used a feasible strategy to study population genetics in forest trees that combined expression patterns and association studies to enhance the understanding of the functions of miRNA and to dissect the allelic interactions of an miRNA and its targets in *P. tomentosa*.

## Materials and Methods

### The Association Population and Phenotypic Data

#### Association Population

An association population of 435 unrelated individuals of *P. tomentosa* was used for SNP-based association analysis, which were randomly selected in a clonal arboretum (Du et al., 2012). This arboretum, located in Guan Xian County, Shandong Province of China (36°23′N, 115°47′E), contained 1,047 unrelated individuals that were collected from a natural distribution of *P. tomentosa* in 1982.

#### Phenotypic Data

We measured 10 quantitative traits from each of the 435 individuals, seven wood-property traits and three growth traits. The wood property traits were α-cellulose content (CC), holocellulose content (HC), hemicellulose content (HeC), lignin content (LC), fiber length (FL), fiber width (FW), and microfiber angle (MFA). The growth traits were diameter at breast height (DBH), tree height (H), and stem volume (V). The detailed measurements and phenotypic variation of these 10 traits were described by Du et al. (2014).

### Identification and Isolation of *Pto-MIR475b* and Its Target Genes

To clone the full-length sequence of *Pto-MIR475b* in *P. tomentosa*, we used gene-specific primers based on the primary sequence of *Ptc-MIR475b* from *P. trichocarpa*, which contained the pre-miRNA region of the *Pto-MIR475b* sequence and 1,000 bp of flanking region on each side.

The putative target genes of Pto-miR475b were predicted by psRNATarget^1^ using 3,000 complementary DNA (cDNA) sequences from the mature xylem cDNA library of *P. tomentosa*. In addition, degradome sequencing was performed to verify the psRNATarget results, which identified *Pto-PPR1, Pto-PPR2, Pto-PPR3*, and *Pto-PPR4* as the putative target genes of Pto-miR475b. Next, we isolated the cDNAs of these candidate target genes from the *P. tomentosa* mature xylem cDNA library.

The total RNA from six tissues (leaf, shoot apex, phloem, cambium, developing xylem, and mature xylem) from *P. tomentosa* were pooled together in equal amounts. The pooled RNA samples were used to build degradome libraries for degradome sequencing according to Zhou et al. (2010). Briefly, T4 RNA ligase (Ambin) was used to add a 5′ adapter to the cleavage products which possess a free 5′ phosphate on their 3′ termini. The ligated products were purified and reverse transcribed with oligo(dT) primers using SuperScript II RT (Invitrogen). The cDNA library was amplified for six cycles (94°C for 30 s, 60°C for 20 s, and 72°C for 3 min). The PCR products were digested with *Mme*I and ligated to a double-stranded DNA adapter using T_4_ DNA ligase. The products were amplified (94°C for 30 s, 60°C for 20 s, and 72°C for 20 s) and gel purified. Finally, the purified products were used for sequencing-by-synthesis with Illumina HiSeq2000. The miRNA cleavage sites were identified with the CleaveLand pipeline (Addo-Quaye et al., 2009) based on the *P. trichocarpa* genome transcripts (v 3.0) (SRX1447192).

### Expression of *Pto-MIR475b* and Its Target Genes Using Real-Time Quantitative PCR (RT-qPCR)

Total RNAs were extracted from eight different tissues: root, shoot apex, cambium, developing xylem, mature xylem, phloem, mature leaf, and young leaf. All samples were collected from a 1-year-old *P. tomentosa* clone, “LM50,” and stored in liquid nitrogen until RNA extraction. The total RNAs were extracted using the Plant Qiagen RNeasy kit (Qiagen China, Shanghai) following the manufacturer’s instructions and RNase-free DNase (Qiagen) was used to purify the total RNA. The total RNAs were reverse transcribed into cDNA with the Reverse Transcription System (Promega Corporation, Madison, WI, United States). RT-qPCR was performed on a 7500 Fast Real-Time PCR System using SYBR Premix Ex Taq according to the manufacturer’s protocol. The specific primers for each gene for qPCR (Supplementary Table S1) were designed by Primer Express 5.0 software (Applied Biosystems). The expression levels were normalized to poplar *Actin* (Accession number: EF145577). All reactions were performed in three technical and biological repetitions, and differential reactions across classes were tested by ANOVA (Analysis of Variance) as described by Song et al. (2013). The qPCR amplification program was as follows: initial denaturation at 94°C for 5 min; followed by 40 cycles of 94°C for 30 s, 58°C for 30 s, and 72°C for 30 s; and a final melting curve of 70–95°C.

### SNP Discovery and Genotyping

To identify SNPs within *Pto-MIR475b* and its four target genes, we randomly selected 40 unrelated individuals from the association population. We designed the primers based on the cDNA sequences of these genes and amplified the genomic DNA from these 40 individuals by PCR. The PCR products were purified and sequenced for subsequent analysis. Gene cloning and purification was performed according to Zhang et al. (2011). To find the SNPs, the genomic sequences of *Pto-MIR475b, Pto-PPR1, Pto-PPR2, Pto-PPR3,* and *Pto-PPR4* from the 40 individuals were analyzed and aligned with MEGA 5.0. The 435 individuals, composed the association population, were provided for genotyping all common SNPs [minor allele frequency (5%)] from *Pto-MIR475b* and four targets according to the methods described by Du et al. (2013).

The genomic sequences of *Pto-MIR475b, Pto-PPR1, Pto-PPR2, Pto-PPR3,* and *Pto-PPR4* in the 40 unrelated individuals of *P. tomentosa* have been deposited in GenBank under the accession numbers KY619249–KY619288, KY619085–KY619124, KY619126–KY619165, KY619167–KY619206, and KY619208–KY619247, respectively.

### Transcript Analysis of the SNP Genotypes

Single-nucleotide polymorphisms occurring in the pre-miRNA region of the miRNA gene might affect the secondary structure and expression level of the corresponding miRNA. Here, we predicted the secondary structure of the normal and mutated sequences of Pto-miR475b using RNAfold^2^. Using the methods described above, we selected 10 individuals for each SNP genotype from the association population and conducted RT-qPCR using RNA from mature xylem to test whether the expression levels of *Pto-MIR475b* and its four target genes varied in the different SNP genotypes. The different expression levels across the different SNP genotypes were tested using ANOVA.

### Data Analysis

#### Nucleotide Diversity Analysis and Linkage Disequilibrium Test

##### Nucleotide diversity analysis

DnaSP program version 5.10 (Librado and Rozas, 2009) was used to evaluate nucleotide diversity, which estimated by π the average number of pair-wise differences per site between sequences, and by 𝜃_w_ the average number of segregating sites per site (Watterson, 1975; Nei, 1987).

##### Linkage disequilibrium (LD) test

The software package TASSEL Ver. 2.0.1^3^ was used to calculate the squared correlation of allele-frequencies (*r*^2^) between pairs of SNPs in *Pto-MIR475b* and its target genes. To assess the pattern of LD in the sequenced region of *Pto-MIR475b* and its target genes, the decay of LD with physical distance (base pairs) within each common SNP was estimated in 10^5^ permutations of the genotype data by non-linear regression, and singletons were excluded in the LD analyses (Remington et al., 2001).

#### Association Analysis

##### Single SNP-based association analysis

In the association population, the mixed linear model (MLM), which controls for kingship and population structure, in the software package TASSEL Ver. 2.0.1^4^ was used to perform all trait–SNP association analyses between 521 SNPs and 10 traits with 10^4^ permutations, QVALUE software in R (Storey et al., 2004) was used to correct for multiple testing with the positive false discovery rate (FDR) method (Storey and Tibshirani, 2003). The MLM is expressed as: *y* = *X***β** + *S***α** + *Q***v** + *Z***u** + **e**, where these letters as a vector, and expressed as below: *y*, phenotypic effects; **β**, fixed effect (except to SNP or population effects); **α,** SNP effects; **v,** population effects; **u,** polygene background effects; **e,** residual effects; *Q*, a matrix from STRUCTURE relating *y* to *v*; and *X*, *S*, and *Z* are incidence matrices of 1s and 0s linking y to **β**, **α**, and **u**, respectively. The variances of the random effects are Var (**u**) = 2*KV*_g_, and Var (**e**) = *RV*_R_, where *K* is an n^∗^n matrix in which the elements are relative kinship coefficients that define the degree of genetic covariance between any two individuals; *R* is an n^∗^n matrix where the elements in off-diagonal are 0 and the elements in diagonal are the reciprocal of the number of observations from each phenotypic data point. *V*_g_ is genetic variance and *V*_R_ is the residual variance (Yu et al., 2006). In our study, we used the Q and K matrix described by Du et al. (2012). The significance levels of the *P*-value and *Q*-value for association analysis results were 0.01 and 0.1, respectively.

##### Haplotype-based association analysis

The haplotype trend regression (HTR) software was used to conduct haplotype association with growth and wood properties of *P. tomentosa*. We used 1000 permutation tests to calculate the significant levels of the haplotype-based associations and used the positive FDR in Q-VALUE to correct the multiple testing.

##### Modes of gene action

To quantify the modes of gene action, we calculated the ratio of dominance to additive effects (d/a) as described by Wegrzyn et al. (2010). If the absolute values of the ratio (|d/a|) were in the range 0.50 < |d/a| < 1.25, the gene action of the marker was partial or complete dominance. Values in the range |d/a| ≤ 0.5 were additive effects, and values in the range |d/a| > 1.25 were under- or over-dominance.

##### Multi-SNP epistasis analysis

Multifactor Dimensionality Reduction 3.0.2 (MDR 3.0.2) (Hahn et al., 2003) was applied to detect and characterize non-additive interactions (such as epistatic effects) among the SNPs. In this software, the RelifF algorithm was used to improve the reliability of probability approximation by filtering all unlinked SNPs (*r*^2^ < 0.1 or different genes), and provide the best five loci for each trait. Finally, an entropy-based measure was used to detect significant interactions between SNP–SNP pairs and calculate information gains (IGs) to evaluate epistatic effects.

## Results

### Identification of *Pto-MIR475b* and Its Four Target Genes

**FIGURE 1:**
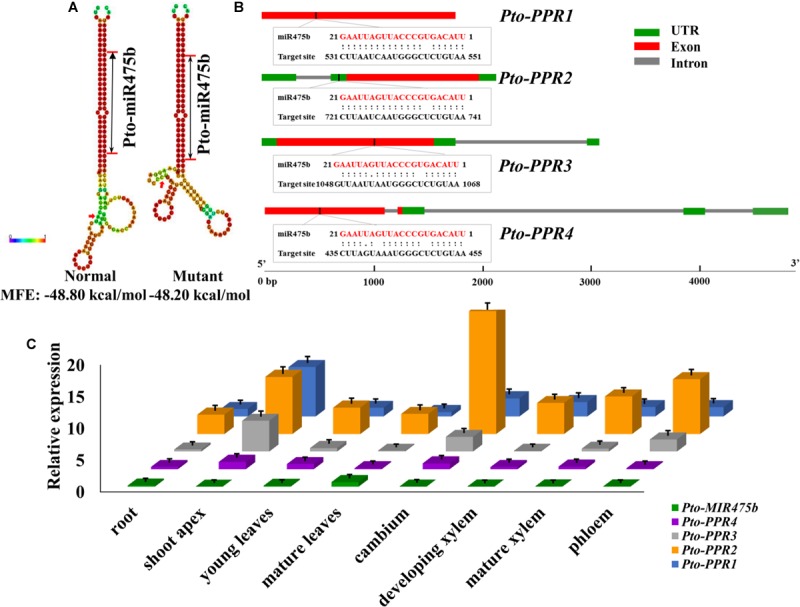
Identification of Pto-miR475b and its four target genes (*Pto-PPR1, Pto-PPR2, Pto-PPR3,* and *Pto-PPR4*). **(A)** The secondary structures of Pto-miR475b affected by the SNP in the precursor region, and the change in the minimum free energy (MFE). **(B)** The gene structures of four target genes and the binding site of Pto-miR475b. **(C)** The expression pattern of *Pto-MIR475b* and its four targets in eight tissues: root, shoot apex, young leaves, mature leaves, cambium, development xylem, mature xylem, and phloem, conducted by RT-qPCR with *Actin* as the internal control.

**FIGURE 2:**
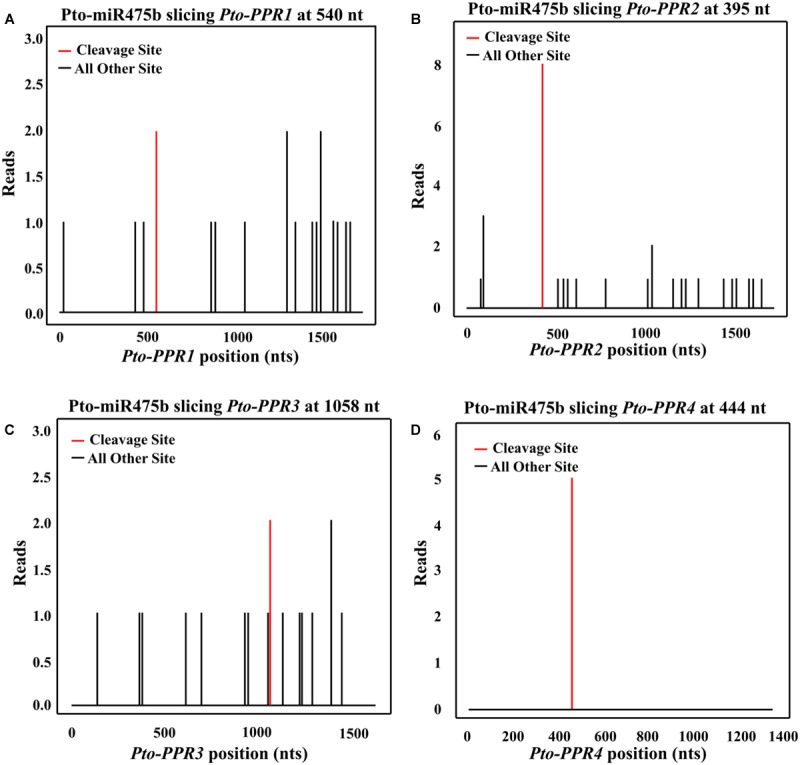
The potential cleavage sites in *Pto-PPR1* **(A)**, *Pto-PPR2* **(B)**, *Pto-PPR3* **(C)**, and *Pto-PPR4* **(D)**. The Pto-miR475b cleavage sites in the four target genes were identified by degradome sequencing. The red vertical bars indicate the most likely cleavage sites.

We isolated the primary sequence of *Pto-MIR475b* based on the sequence of *Ptc-MIR475b* in miRBase and found that miR475b is a poplar-specific miRNA. The sequence contained 21 nt of the mature miRNA region of *Pto-MIR475b*, 136 nt of the pre-miRNA region, and 1,000 nt of flanking sequence on each side of the pre-miRNA region. The secondary structure showed a typical stem-loop structure, which is the major characteristic of miRNAs (**Figure 1A**). The 21 nt mature sequence of Pto-miR475b and the mature xylem cDNA library of *P. tomentosa* were analyzed in psRNATarget to predict its target genes, which identified 78 candidate targets. Degradome sequencing, a high-throughput sequencing method used to determine the sequence of RNA ends, by identifying miRNA cleavage sites (Addo-Quaye et al., 2008; Zhou et al., 2010). We performed degradome sequencing to detect the most likely cleavage sites in the genes, which identified four putative target genes of Pto-miR475b. Then we isolated cDNA sequences of these four target genes from a cDNA library from mature xylem of *P. tomentosa*. By BLAST searches against the *P. trichocarpa* genome sequence (Tuskan et al., 2006), we found high similarity in the full-length cDNA sequences of the four targets to Potri.006G242200 (97%), Potri.006G271200 (98%), Potri.011G057900 (98%), and Potri.013G130600 (98%) (Supplementary Table S2). All four of the genes encode pentatricopeptide repeat (PPR) superfamily proteins; therefore, we named them *Pto-PPR1*, *Pto-PPR2*, *Pto-PPR3,* and *Pto-PPR4*. Sequence analysis showed that the *Pto-PPR1* (Accession number: KY619125)*, Pto-PPR2* (Accession number: KY619166), *Pto-PPR3* (Accession number: KY619207), and *Pto-PPR4* (Accession number: KY619248) were 1,794, 1,795, 1,915, and 1,900 bp in length, and encoded proteins of 597, 403, 478, and 384 aa, respectively (**Figure 1B**). In addition, the degradome results showed that Pto-miR475b cleaved *Pto-PPR1* at 540 nt, *Pto-PPR2* at 395 nt, *Pto-PPR3* at 1,058 nt, and *Pto-PPR4* at 444 nt (**Figure 2**).

### Tissue-Specific Expression Patterns Revealed a Negative Correlation between *Pto-MIR475b* and Its Four Target Genes

RT-qPCR was used to detect the relative expression levels of *Pto-MIR475b* and its four target genes in eight different tissues of *P. tomentosa*. *Pto-MIR475b* and its four target genes were expressed in all examined tissues with varied transcript abundance (**Figure 1C**). The expression level of *Pto-MIR475b* was highest in mature leaves, followed by the root, and lowest in the shoot apex. By contrast, *Pto-PPR1, Pto-PPR3*, and *Pto-PPR4* showed the highest expression in the shoot apex, and *Pto-PPR2* had the highest expression in the cambium. *Pto-PPR1* and *Pto-PPR4* had the lowest expression in mature leaves, whereas *Pto-PPR2* and *Pto-PPR3* showed the lowest expression in the root and developing xylem. Moreover, the correlation coefficient (*r*) between *Pto-MIR475b* and its four target genes ranged from -0.447 to -0.411, which showed a negative correlation and suggested that Pto-miR475b potentially negatively regulates the four target genes.

### High Nucleotide Diversity and Rapidly Decayed LD in *Pto-MIR475b* and Its Four Target Genes

To identify SNPs in *Pto-MIR475b* and its four target genes, we sequenced these five genes in 40 randomly selected individuals of the association population of *P*. *tomentosa*. For *Pto-MIR475b*, we identified 19 SNPs with a frequency of 1/112 bp (π = 0.00208, 𝜃_w_ = 0.00209). No SNPs were identified in the mature miRNA region, indicating that this region was extremely conserved. In the precursor region, one SNP (*Pto-MIR475b*-SNP19) was identified with a frequency of 1/135 bp (π = 0.00137, 𝜃_w_ = 0.00174) (**Table 1** and Supplementary Table S4). This SNP altered the stem-loop structure of Pto-miR475b and the minimum free energy of the predicted secondary structure (**Figure 1A**). For the target genes, we identified 542 SNPs with a frequency range of 1/103 bp (*Pto-PPR3*) to 1/36 bp (*Pto-PPR1*). The nucleotide diversity demonstrated that the four target genes were under different selective pressure (Zhang et al., 2011). We calculated the average synonymous diversity (*d_S_*) and the non-synonymous diversity (*d_N_*) for the coding region of three of the target genes and the ratio of *d_N_/d_S_* < 1 (*Pto-PPR1,* 0.427; *Pto-PPR2,* 0.759; *Pto-PPR3,* 0.062) indicated that the non-synonymous sites experienced purifying selection (**Table 1** and Supplementary Table S4). *Pto-PPR4* had no SNP mutations in the exon region, suggesting a highly conserved coding region.

**Table 1:** Summary of single-nucleotide polymorphisms in *Pto-MIR475b* and the four target genes.

Gene	Region	Length (bp)	No. of polymorphic sites	No. of common SNPs	Percentage of polymorphisms (%)	Nucleotide diversity	
						π	𝜃w
*Pto-MIR475b*							
	pre-miR475b	135	1	1	0.74	0.00137	0.00137
	pri-miR475b	2135	19	19	0.90	0.00208	0.00208
*Pto-PPR1*							
	Synonymous	402.04	11	11	2.72	0.00869	0.00643
	Non-synonymous	1388.96	15	15	1.08	0.00371	0.00254
	Total silent^a^	4405.04	142	142	3.22	0.01157	0.00758
	Total^b^	5794	157	157	2.71	0.0097	0.00645
*Pto-PPR2*							
	Synonymous	271.03	11	10	4.06	0.015	0.00954
	Non-synonymous	937.98	28	21	3.00	0.01139	0.00702
	Total silent^a^	5191.02	128	113	2.47	0.00912	0.0058
	Total^b^	6130	156	144	2.54	0.00946	0.00598
*Pto-PPR3*							
	Synonymous	310.83	2	2	0.64	0.0021	0.00151
	Non-synonymous	1123.17	1	1	0.09	0.00013	0.00021
	Total silent^a^	6004.83	68	67	1.13	0.00309	0.00266
	Total^b^	7128	69	68	0.97	0.00262	0.00228
*Pto-PPR4*							
	Synonymous	259.5	0	0	0	0	0
	Non-synonymous	892.5	0	0	0	0	0
	Total silent^a^	7952.5	141	133	1.77	0.00599	0.00417
	Total^b^	8845	141	133	1.59	0.00538	0.00375

^a^Total silent: synonymous sites plus polymorphic sites in non-coding regions of genes.

To perform the association analysis, we selected 521 common SNPs (minor allele frequencies > 5%) from *Pto-MIR475b* and its four target genes that were genotyped in 435 unrelated individuals of *P. tomentosa*. Nineteen common SNPs were identified in *Pto-MIR475b*, with one located in the pre-miRNA region. Of the four target genes, 88.05% of the total 502 SNPs were found in the non-coding regions: the flanking region (2,000 bp of the flanking sequence of the 5′-UTR and 3′-UTR, 61.75%), 5′-UTR (2.59%), intron region (19.32%), and 3′-UTR (4.38%) (Supplementary Table S3).

The squared allelic correlation coefficient (*r*^2^) between each common SNP pair was calculated to evaluate the overall patterns of LD for *Pto-MIR475b* and its four target genes (**Supplementary Figure S1**). The non-linear regression results showed that LD decayed rapidly with *r*^2^ values and dropped to 0.1 within approximately 300 bp for *Pto-MIR475b*, and within approximately 400 to 2,500 bp for each target gene, indicating that the LD of *Pto-MIR475b* and its four target genes does not extend over the entire genes.

### Dissection of Significant SNP–Trait Associations in *P. tomentosa*

**FIGURE 3:**
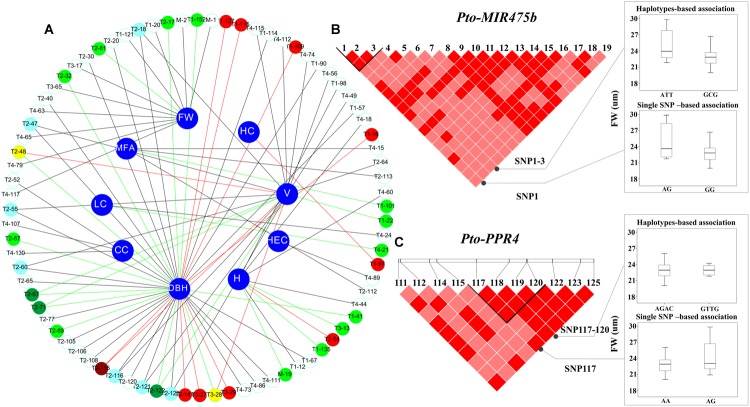
The single SNP-based and haplotype-based associations in the natural population. **(A)** The single SNP-based associations between significant SNPs from *Pto-MIR475b* and its four targets and the traits. M, T1, T2, T3, and T4 indicate *Pto-MIR475b*, *Pto-PPR1, Pto-PPR2, Pto-PPR3,* and *Pto-PPR4,* respectively. The red lines represent additive effects, the green lines represent partial or complete dominance, and the dark lines represent under- or over-dominance. In addition, the white, light red, and light green circles represent under- or over-dominance, additive, and partial or complete dominance effects, respectively; the light blue, deep red, and deep green circles represent a SNP associated with two traits with the same mode of gene action, and the yellow circles represent a SNP within additive effects and partial or complete dominance. **(B)** The genotypic effect for the significant haplotypes of *Pto-MIR475b*_SNP1-3, along with the genotypic effect for the single locus of *Pto-MIR475b*_SNP1 consistent with single SNP-based association. **(C)** The genotypic effects for the *Pto-PPR4*_SNP117-120 and *Pto-PPR4*_SNP117.

To test the effects of the SNPs on tree growth and wood properties, we conducted association analysis, which detected 93 significant associations (*P* < 0.01, *Q* < 0.1) representing 80 unique SNPs in *Pto-MIR475b* and the four target genes with nine traits. The phenotypic variance (*R*^2^) explained by each SNP ranged from 1.50 to 22.57% (average *R*^2^= 12.73%) (**Figure 3A**, **Table 2**, and Supplementary Table S5). For *Pto-MIR475b*, three SNPs were significantly associated with two traits (H and FW), which suggested that *Pto-MIR475b* is potentially involved in tree growth and wood formation. *Pto-MIR475b*_SNP1 and *Pto-MIR475b*_SNP2 were located in the flanking regions and associated with FW with different phenotypic contributions (*R*^2^ of 16.25 and 16.97%, respectively). As expected, the SNP in the precursor region, *Pto-MIR475b*_SNP19, was significantly associated with *H* with an *R*^2^ of 2.62%, indicating that *Pto-MIR475b* may play important roles in regulating tree height. The other 77 SNPs were distributed amongst the four target genes and were significantly associated with nine traits. Of these SNPs, 10.39% were located in coding regions. For example, *Pto-PPR2*_SNP64, located in the coding region of *Pto-PPR2*, caused a non-synonymous mutation of Phe to Ser, which was associated with *V* with an *R*^2^ of 15.14%. *Pto-PPR1*_SNP109, located in the promoter region of *Pto-PPR1*, had the largest phenotypic contribution with DBH. Interestingly, we found that 13 of the 77 SNP markers were significantly associated with two traits (DBH and V). For instance, *Pto-PPR2*_SNP67 caused a missense mutation (His to Tyr), and associated with DBH and *V* with an *R*^2^ of 17.96 and 12.19%, respectively.

**Table 2:** Summary of significant SNPs within *Pto-MIR475b* and the four targets associated with growth and wood properties in the association population of *P. tomentosa.*

Traits	Number of association	Number of SNPs					Range of *R*^2^ (%)	Average of *R*^2^ (%)
		*Pto-MIR475b*	*Pto-PPR1*	*Pto-PPR2*	*Pto-PPR3*	*Pto-PPR4*		
CC (%)	2	–	–	–	–	2	14.67 ∼ 17.97	16.32
DBH (cm)	43	–	10	27	3	3	1.50 ∼ 22.57	13.75
FW (μm)	11	2	2	1	2	4	11.31 ∼ 17.07	14.05
H (m)	9	1	3	1	1	3	1.72 ∼ 6.40	3.43
HC (%)	1	–	1	–	–	–	16.45	16.45
HEC (%)	1	–	–	–	–	1	11.17	11.17
LC (%)	2	–	–	–	–	2	11.12 ∼ 11.71	11.47
MFA (∘)	4	–	1	2	–	1	11.18 ∼ 15.85	13.54
V (m^3^)	20	–	1	13	1	5	12.19 ∼ 18.83	13.54
Total	80	3	18	32	6	21	1.50 ∼ 22.57	12.74

In addition, we used the ratio of dominance (d) to additive (a) effects to quantify the modes of gene action for SNP–trait associations. Thirty SNP–trait associations showed additive effects (|d/a| ≤ 0.5). The other associations were divided into two modes of gene action, over- or under-dominance (|d/a| ≥ 1.25, *n* = 59) or partially to fully dominant effects (0.50 < |d/a| < 1.25, *n* = 21). For instance, *Pto-PPR1*_SNP97 showed additive effects on HC (63.88% for GG, 74.05% for GA, 69.36% for AA). The different values of the effect on *V* among the three genotypes of *Pto-PPR2*_SNP64 were significant, suggesting that the mode of gene action was under-dominance (0.87 m^3^ for CC, 0.50 m^3^ for CT, 0.65 m^3^ for TT). *Pto-PPR3*_SNP28 was significantly associated with LC, indicating that the pattern of gene action was a partially to fully dominant effect (26.05 cm for AA, 21.38 cm for AG, 20.57 cm for GG). Interestingly, one SNP can contribute to different traits with the same mode or different modes of gene action. For example, *Pto-PPR2*_SNP115 showed both additive effects on DBH (18.12 cm for GG, 25.58 cm for AG, 20.45 cm for AA) and *V* (0.36 m^3^ for GG, 0.88 m^3^ for AG, 0.53 m^3^ for AA). However, *Pto-PPR2*_SNP48 was significantly associated with DBH and V, but with different modes of gene action, with additive effects on *V* (0.88 m^3^ for AA, 0.39 m^3^ for AC, 0.52 m^3^ for CC) and partially to fully dominant effects on DBH (25.84 m^3^ for AA, 20.21 m^3^ for AC, 19.04 ^3^ for CC) (**Figure 3A**).

### Single SNP–Trait Associations Support the Haplotype-Based Association

We used the HTR model to conduct the association studies. We identified 50 common haplotypes (frequency > 0.05) from 28 high LD blocks (*r*^2^ > 0.75, *P* < 0.001) within *Pto-MIR475b* and its four target genes (**Table 3** and Supplementary Table S6). Each gene contained 3–5 blocks, and each block had 1–2 haplotypes. We detected 50 significant associations between 39 haplotypes from 28 blocks and seven traits (CC, LC, HC, HeC, FW, FL, H) with different *R*^2^ values ranging from 0.15% to 23.17% (average *R*^2^ of 6.48%). Seven haplotypes were associated with multiple traits. For example, the haplotype C-T from block *Pto-PPR3*_SNP9-11 simultaneously associated with LC, AC, HeC, and HC with different *R*^2^ values ranging from 3.19 to 6.80%. The other haplotype (T-A) from this block associated with HeC with an *R*^2^ of 4.28%, indicating that the gene has pleiotropic effects. In addition, we found that 20 haplotypes from 18 blocks in *Pto-MIR475b* and its four targets (three come from *Pto-MIR475b,* four from *Pto-PPR1,* five from *Pto-PPR2,* three from *Pto-PPR3,* and five from *Pto-PPR4*) associated with FW with an *R*^2^ ranging from 0.15 to 23.17% (average *R*^2^ of 11.01%). Compared with the single SNP-based association results, we found that six SNPs overlapped in haplotype-based associations. For example, *Pto-MIR475b*_SNP1 and *Pto-MIR475b*_SNP2 significantly associated with FW under the single SNP-based association, and the haplotypes (A-T-T and G-C-G) came from the block (*Pto-MIR475b*_SNP1-3) associated with FW under the haplotype-based association (**Figure 3B**). *Pto-PPR4_*SNP117 and the haplotype (A-G-A-C) came from the block (*Pto-PPR4_*SNP117-120) associated with FW under their respective models. These results indicated that the single SNP-based associations support the results of the haplotype-based associations (**Figure 3C**).

**Table 3:** Summary of significant haplotypes within *Pto-MIR475b* and the four targets associated with growth and wood properties in the association population of *P. tomentosa.*

Gene	Number of LD blocks	Length range of haplotypes^a^	Number of haplotypes	Associated traits	Range of *R*^2^ (%)
*Pto-MIR475b*	3	2–5	6	CC,FW,LC	0.38–18.11
*Pto-PPR1*	7	2–5	8	CC,FW,HeC,LC	1.25–15.81
*Pto-PPR2*	6	2–5	9	CC,FW,H,LC	0.47–23.17
*Pto-PPR3*	7	2–4	10	CC,FW,HC,HeC,LC	0.15–10.36
*Pto-PPR4*	5	3–5	6	CC,FW	1.30–22.10
Total	28	2–5	39	CC,FW,HC,HeC,LC	0.15–23.17

**^a^**Length range of haplotypes: one SNP as a unit.

### The Genotypic Combinations of SNP–SNP Pairs from *Pto-MIR475b* and Its Four Target Genes Have Different Epistatic Effects for Growth and Wood Property

**FIGURE 4:**
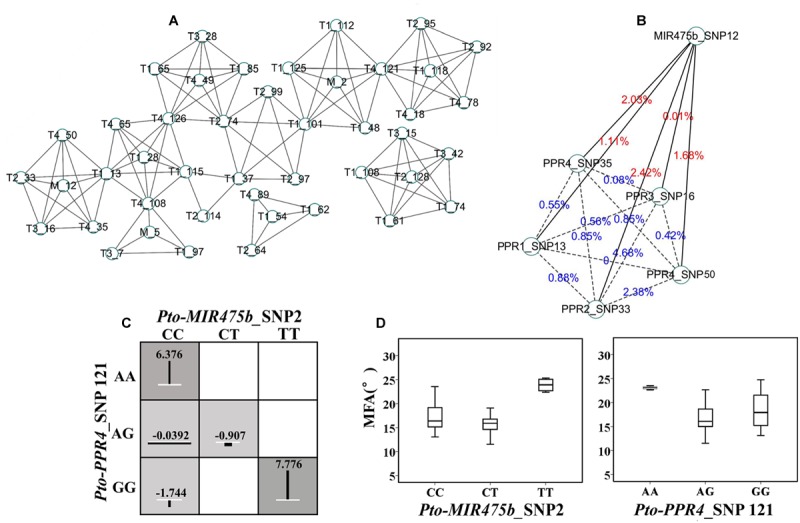
The epistatic network within the SNPs from *Pto-MIR475b* and the four target genes. **(A)** A structural network revealed the epistatic interactions of the SNPs in *Pto-MIR475b* and the four target genes. **(B)** The network of *Pto-MIR475b* and four targets, showing epistatic interactions affecting H. The solid lines represent the epistatic interactions between *Pto-MIR475b* and the four targets, the dashed lines represent the epistatic interactions between gene targets. The values indicate the pairwise epistatic effect. **(C)** Epistatic effects of different genotype combinations for *Pto-MIR475b*_SNP2 and *Pto-PPR4*_SNP121 within MFA. Dark shaded groups showed high interaction values, light shaded groups showed low interaction values. The length of the vertical lines represent the interaction effects of different genotype combinations relative to the mean values of the three genotypes. White lines represent the average effects of different genotypes. **(D)** The genotypic effects for the two SNPs analyzed in **(C)** above.

We used MDR 3.0.2 to detect and characterize the epistatic interactions. We detected 115 significant SNP–SNP associations representing 45 unique SNPs from *Pto-MIR475b* and its four target genes with 10 traits (*P* < 0.01) (**Figure 4A**). In addition, single SNP effects ranged from 0.1 to 8.37%, and the pairwise SNP–SNP effects ranged from 0 to 9.81%. For these SNP–SNP pairs, 11.30% showed an interaction between the *Pto-MIR475b* and its targets, 63.48% showed an interaction between the target genes, and the remaining 25.22% showed SNP–SNP interactions on the same genes (**Table 4** and Supplementary Table S7). Moreover, SNPs in the *Pto-MIR475b* simultaneously showed epistatic effects with SNPs in the four putative gene targets. For instance, *Pto-MIR475b*_SNP12 formed five interactions with H, which were from four putative target genes with epistatic effects ranging from 0.01 to 2.42% (**Figure 4B**). In addition, we used IG to estimate the epistatic effects of the SNP–SNP pairs. IGs ranged from -0.0808 to 0.0371, and 11.30% of the SNP–SNP pairs were positive IGs. The negative IGs indicated redundancy, which means a partial overlap of function in a number of genes involved in the same processes (Pickett and Meeks-Wagner, 1995; Hu et al., 2013).

**Table 4:** Summary of all significant SNP pairs associated with each trait under the epistasis model in the association population of *P. tomentosa.*

Traits	Number of associations	Number of SNPs					Range of interaction effect (%)	Range of IGs (%)
		*Pto-MIR475b*	*Pto-PPR1*	*Pto-PPR2*	*Pto-PPR3*	*Pto-PPR4*		
CC (%)	15	–	2	1	1	2	0 ∼ 3.17	-6.95 ∼ 0.93
DBH (cm)	3	–	2	1	–	–	0.05 ∼ 2.38	-1.82 ∼-0.1
FL (mm)	15	–	1	2	–	3	0.23 ∼ 9.81	-4.1 ∼ 2.45
FW (μm)	15	–	3	–	–	3	0 ∼ 2.38	-6.94 ∼ 0.64
H (m)	15	1	1	1	1	2	0 ∼ 4.68	-5.91 ∼ 1.21
HC (%)	6	1	–	1	1	1	0 ∼ 4.45	-8.08 ∼-1.09
HEC (%)	6	–	2	1	–	1	0.52 ∼ 7.59	-2.78 ∼-1.04
LC (%)	15	–	3	1	2	–	0.08 ∼ 6.83	-7.69 ∼ 0.51
MFA (∘)	15	1	4	–	–	1	0.16 ∼ 6.83	-6.63 ∼ 3.71
V (m^3^)	10	–	2	3	–	–	0.33 ∼ 2.3	-2.39 ∼ 0.93
Total	115	3	17	9	5	10	0 ∼ 9.81	-8.08 ∼ 3.71

For these 115 significant SNP–SNP pairwise associations, we found that the SNPs in *Pto-MIR475b* had epistatic effects with several SNPs from multiple genes on the same phenotype. Therefore, we constructed an interaction network to describe the results. For example, *Pto-MIR475b*_SNP12 and five SNPs from *Pto-PPR1, Pto-PPR2, Pto-PPR3,* and *Pto-PPR4* formed pairwise interactions with H (**Figure 4B**). All 13 pairs showed negative IGs (-0.0663 to -0.0127) and interaction effects for the associated traits of 0 to 3.6%; and presented 13 unique SNPs, each showing single effects for the associated traits (0.01 to 5.74%).

To explore the different effects of single genotypes and genotypic combinations of SNP–SNP pairs, we detected that *Pto-MIR475b*_SNP2 and *Pto-PPR4*_SNP121 formed an epistatic interaction on MFA, and we calculated the average phenotypic values of each genotype (**Figures 4C,D**). For *Pto-MIR475b*_SNP2, the difference between the average phenotypic values of three genotypes (CC, CT, TT) and the total average of phenotypic values (17.557°) ranged from -1.512° (CT, 16.044°) to 7.170° (TT, 24.727°), while for *Pto-PPR4*_SNP121 (AA, AG, GG) the values ranged from -0.177° (AG, 17.379°) to 5.771° (23.327°). The differences between the genotypic combinations and the total average phenotypic values ranged from -1.744° (CC-GG) to 7.776° (TT-GG), which were significantly higher than the phenotypic values of the genotypes of single SNP markers. The significant differences between the average phenotypic values of genotypic combinations and single genotypes of two SNP markers verified the epistatic interactions, which also verified the interactions between *Pto-MIR475b* and its four target genes.

### Transcript Analysis of Significant SNP Genotypes

To explore the roles of the SNP (*Pto-MIR475b*_SNP19) in the pre-miRNA region on the expression level of *Pto-MIR475b*, we used RT-qPCR to detect the relative expression in the 20 unrelated individuals (10 individuals each from the AA group and AC group). *Pto-MIR475b*_SNP19 significantly affected the transcript levels of *Pto-MIR475b*, with the highest level in the AC group (1.949 ± 0.078, arbitrary units normalized to the control), and the lowest level in the AA group (0.216 ± 0.008) (**Supplementary Figure S2**). To test whether the altered expression of *Pto-MIR475b* would affect the expression of the targets, we detected the expression levels of the four target genes in these 20 unrelated individuals. Compared with the average relative expression levels of the different genotype groups, we found that *Pto-MIR475b* displayed an inhibited action on the expression levels with its four targets. The expression level of *Pto-MIR475b* was higher for the AC genotype than the AA genotype, but the corresponding expression levels of the four target genes were lower in the AC genotype than AA genotype (**Supplementary Figure S2**).

## Discussion

### The Characterizations and Functions of *Pto-MIR475b*

MiRNAs are non-coding RNAs with important regulatory functions, and play a crucial role in various biological processes such as plant development and stress tolerance (Chen, 2005; Jones-Rhoades et al., 2006). However, the function of miRNAs can be affected by allelic variations. SNPs may alter the function of miRNAs by changing the pre-miRNA secondary structure and affecting transcript abundance, which can lead to phenotypic variations (Sun et al., 2009; Xu et al., 2013). For instance, one SNP in the pre-miRNA region of *Pto-MIR160a* altered the number of loops in its secondary structure and was significantly associated with tree growth in *P. tomentosa* (Tian et al., 2016). In our study, we identified a common SNP (*Pto-MIR475b*_SNP19) in the precursor region of *Pto-MIR475b*, which significantly altered the stem-loop structure and changed the minimum free energy of the secondary structure of Pto-miR475b (**Figure 1A**), which affected its transcription and may impair the generation of the mature miRNA (Duan et al., 2007; Bensen et al., 2013). Furthermore, we tested the transcript abundance in different SNP genotypes of *Pto-MIR475b*_SNP19. We found significant differences between the different genotypes (**Supplementary Figure S2**), suggesting that *Pto-MIR475b*_SNP19 can affect the transcription of *Pto-MIR475b*, which supported the idea that a SNP in the pre-miRNA region can have a regulatory function. We also detected an opposite expression pattern between *Pto-MIR475b* and its four targets in the different genotypes of *Pto-MIR475b*_SNP19, suggesting that the transcript abundance of *Pto-MIR475b* will affect the expression of its target genes. Moreover, the results of the single SNP-based association study showed that *Pto-MIR475b*_SNP19 significantly associated with H with an *R*^2^ of 2.62%, and showed partially to fully dominant effects. These association results also supported the concept that SNPs may play an important regulatory role in miRNA biogenesis (Duan et al., 2007; Sun et al., 2009).

In addition, several studies have reported that the flanking sequence may affect the efficiency of cleavage of the primary transcript to form the pre-miRNA. For example, Zeng and Cullen (2005) reported that pre-miRNA processing *in vitro* requires flanking sequences attached to the pre-miRNA stem-loop structure. According to their distribution pattern and frequency, SNPs very likely occur in these long flanking regions (Cao et al., 2011). Here, we identified 18 common SNPs (MAF > 5%) in the flanking region of *Pto-MIR475b* with nucleotide diversity of π = 0.00208, which was higher than the precursor region of Pto-miR475b (π = 0.00137). No SNPs were found in the mature miRNA region, indicating that the mature miRNA region is highly conserved (**Table 1**). Through alignment with other species, we found that miR475b is a *Populus*-specific miRNA, and is completely conserved compared with the orthologous sequence in *P. trichocarpa*.

Next, the results of the single SNP-based associations and haplotype-based associations of 18 common SNPs in the flanking region revealed that two SNPs (*Pto-MIR475b*_SNP1 and *Pto-MIR475b*_SNP2) were significantly associated with FW, indicating that SNPs in the flanking region may contribute to phenotypic variation. Under the haplotype-based association, we identified eight associations in six haplotypes with three traits. For instance, the haplotypes (A-T-T, G-C-G) in the *Pto-MIR475b*_SNP1-3 block associated with FW, and the single SNP-based associations showed that *Pto-MIR475b*_SNP1 and *Pto-MIR475b*_SNP2 both associated with FW. These results indicated that the haplotype association was supported by the single SNP-based association, suggesting that multiple SNPs composed a haplotype and can lead to phenotypic variation (**Figure 3B**). These haplotype-based association studies illuminated the allelic effects of SNPs in *Pto-MIR475b*, indicating that *Pto-MIR475b* may play an important regulatory role in tree growth and wood formation.

### SNPs in *Pto-MIR475b* and Its Four Gene Targets Associated with Growth and Wood Properties

Through the single SNP-based and haplotype-based associations, we detected 93 significant SNP–trait associations and 50 significant haplotype–trait associations for *Pto-MIR475b* and its four targets, respectively, and the 10% haplotype results were supported by the single SNP-based associations, indicating that these five genes may have similar roles in tree growth and wood formation (**Tables 2**, **3**). For *Pto-MIR475b*, three common SNPs significantly associated with H and FW. We found that 17 SNPs in *Pto-PPR1, Pto-PPR2, Pto-PPR3,* and *Pto-PPR4* were significantly associated with H and FW (average *R*^2^= 8.80%). Additionally, we observed that haplotypes in *Pto-MIR475b* and its targets associated with the same traits. For instance, three haplotypes from *Pto-MIR475b* associated with FW, and 17 haplotypes from the four target genes associated with FW. These results suggested that *Pto-MIR475b* and its four gene targets are involved in the same regulatory pathway for tree growth and wood formation, and the multiple SNPs associated with various traits revealed the pleiotropy of the genes (**Figure 3A**), and demonstrated the allelic interactions between *Pto-MIR475b* and four putative targets.

The functions of miRNAs mainly depend on their target genes (Chen, 2005). Thus, to improve our understanding of Pto-miR475b, we dissected the effect of allelic variation in its four targets. The single SNP-based association studies showed that four SNPs (*Pto-PPR2*_SNP64, *Pto-PPR2*_SNP65, *Pto-PPR2*_SNP67, and *Pto-PPR2*_SNP77) caused non-synonymous mutations and associated with DBH and V, indicating that *Pto-PPR2* may be involved in tree growth (Supplementary Table S5). *Pto-PPR1*_SNP109, located in the promoter region of *Pto-PPR1*, had the largest phenotypic contributions (22.57%) to DBH, indicating that *Pto-PPR1* may play a major role in tree growth. We also observed that a single SNP significantly associated with two traits. Thirteen SNPs associated with DBH and *V* with different *R*^2^ values, and the majority of the SNPs occurred in *Pto-PPR2* (12 SNPs), with only one in *Pto-PPR3*. *Pto-PPR2*_SNP67 caused a missense mutation and associated with DBH and *V* with an *R*^2^ of 17.96 and 12.19%, respectively, with partially to fully dominant effects for both traits, indicating the importance of the SNP marker and the pleiotropy of the gene. Moreover, the target genes were highly expressed in the shoot apex and cambium (**Figure 1C**), and supported the idea that *PPRs* might play a crucial role in the growth process (Hu et al., 2016). PPR proteins are a superfamily of nuclear-encoded proteins that are transported into the mitochondria or chloroplast and perform their functions by RNA splicing, RNA editing, RNA maturation, and translation (Schmitz-Linneweber and Small, 2008; Liu et al., 2016). The *Pto-PPRs* were highly expressed in the actively dividing tissue, which supported the putative roles of *Pto-PPRs* in tree growth. Moreover, our results demonstrated that non-synonymous mutations within *Pto-PPRs* contributed to differences in tree growth and wood formation. In addition, the effect of SNPs in the coding region may alter the post-transcriptional regulation of mitochondria and chloroplast genes through impairing the structure and features of PPR proteins (Barkan and Small, 2014). CRISPR/Cas9 is a relatively new genome editing tool that has been successfully employed in many plants (Feng et al., 2013; Demirci et al., 2017). This tool can be used to explore the functions of the interacting networks of Pto-miR475b and *PPRs* and the effects of SNPs in *Pto-MIR475b* and its target genes.

### The Epistasis Model Dissected the Interaction between *Pto-MIR475b* and the Four Target Genes

The single SNP-based association studies successfully dissected the additive and dominant effects of the SNPs on several complex traits, but quantitative traits are regulated by multiple genes. Therefore, we used epistatic effects to dissect the genetic interactions between SNP–SNP pairs (Mackay, 2014; Wei et al., 2014). Epistasis is a non-linear genetic interaction between two SNP markers, and is an important tool to decipher the genetic effects of SNPs on complex traits (Heidema et al., 2006; Huang et al., 2012). Exploration of the interactions between SNP–SNP pairs will improve the understanding of the long-term response to selection and inbreeding depression (Mackay, 2014). In our study, we evaluated the interactions between SNP–SNP pairs by IGs. We found that 88.7% of 115 significant SNP–SNP pairs showed negative IGs, which suggested that two SNPs carried redundant information (Moore et al., 2006; Hu et al., 2013). SNP–SNP interactions showing positive IGs provide a contribution to phenotypic variation more than the sum of two single SNP effects. Either negative or positive IGs indicate that two SNPs have a similar effect on the same traits.

Epistasis represents the interactions between genes; thus, we used the epistasis model to verify the interactions between miRNA and its targets. For the 115 SNP–SNP pairs, 11.30% showed an interaction between *Pto-MIR475b* and its targets (**Figure 4A**). For example, *Pto-MIR475b*_SNP12 interacted with five SNPs from the four putative target genes, and formed five epistatic interactions with H with pairwise effects ranging from 0.01 to 2.42%, and all IGs were negative, indicating that *Pto-MIR475b* and its four targets interact to co-regulate tree growth and wood formation.

To dissect the epistatic effects of gene interaction on complex traits, we conducted the phenotype of genotype combinations of SNP–SNP pairs. For example, *Pto-MIR475b*_SNP2 and *Pto-PPR4*_SNP121 formed epistatic interactions for MFA with a pairwise effect of 3.6% and an IG of -1.71% (**Figure 4C**). Then we determined the differences between the average phenotypic values of three single genotypes of SNP markers and the total average phenotypic values, and the differences between genotype combinations and total average phenotypic values. The different values of genotype combinations were higher than the single genotypes, indicating the interaction between *Pto-MIR475b* and its four target genes (**Figure 4D**) and suggesting that epistatic interactions of genotype combinations conferred greater effects on the phenotypic variation. In addition, the genotype of *Pto-MIR475b*_SNP2 was CC and the genotype of *Pto-PPR4*_SNP121 was GG, the epistatic effect of the genotype combination (15.21°) for MFA was significantly lower than any single genotype effects (CC: 17.78°; GG: 18.38°), indicating that phenotypic contributions of genotype combinations were less than the single genotype effects. These findings represent examples of epistatic effects and illustrate the allelic interactions of *Pto-MIR475b* and four putative targets for tree growth and wood formation. In addition, recent studies have discovered that miRNA activity is also impaired by endogenous competing RNAs, a phenomenon termed endogenous target mimicry (eTM) (Franco-Zorrilla et al., 2007; Thomson and Dinger, 2016). For example, Wang et al. (2016) revealed a competitive RNA regulatory network in which the long non-coding RNA H19 affected the expression of *FOXM1* by competitively binding with has-miR342-3p in gallbladder cancer. The use of databases, such as PeTMbase (Karakülah et al., 2016), can predict the putative eTMs of miRNA, thus providing a new tool for understanding the regulatory mechanisms of miRNA by building an miRNA–eTM–target network.

## Conclusion

Here, we combined expression profiles and association studies to explore the effects of allelic variation in *Pto-MIR475b* and its four putative target genes. Analysis of expression patterns revealed negative correlations between *Pto-MIR475b* and its four target genes in eight tissues. Single SNP-based and haplotype-based association studies showed that significant SNP markers from five genes were involved in the same biological pathways. The epistasis model demonstrated that *Pto-MIR475b* and its four targets form a complex interactive network affecting phenotypic variation for tree growth and wood formation, and the different genotype combinations of SNP–SNP pairs were further used to dissect the epistatic effects of gene interactions on complex traits.

## Data Archiving Statement

Sequence data in this article has been submitted to GenBank: accession numbers KY619085–KY619288.

## Author Contributions

DZ conceived and designed the experiment; LX and MQ collected the data and conducted statistical analysis; LX wrote the manuscript; MQ, QD, JC, and JX provided valuable suggestions for the manuscript; MQ, QD, and DZ revised the manuscript; DZ obtained funding and is responsible for this article. All authors read and approved the manuscript.

## Conflict of Interest Statement

The authors declare that the research was conducted in the absence of any commercial or financial relationships that could be construed as a potential conflict of interest.

## Footnotes

1 http://plantgrn.noble.org/psRNATarget/

2 http://rna.tbi.univie.ac.at/

3 http://www.maizegenetics.net/

4 http://www.maizegenetics.net/

## Supplementary material

FIGURE S1 Decay of linkage disequilibrium of SNPs within *Pto-MIR475b, Pto-PPR1, Pto-PPR2, Pto-PPR3,* and *Pto-PPR4.*

FIGURE S2 Expression levels of *Pto-MIR475b* and four target genes in the different genotypes of *Pto-MIR475b_SNP19*.
