# Supplementary figures and images for: Allelic Interactions among Pto-MIR475b and Its Four Target Genes Potentially Affect Growth and Wood Properties in Populus
Source: Front Plant Sci. 2017 Jun 21;8:1055. doi: 10.3389/fpls.2017.01055 (PMC5478899; doi:10.3389/fpls.2017.01055)

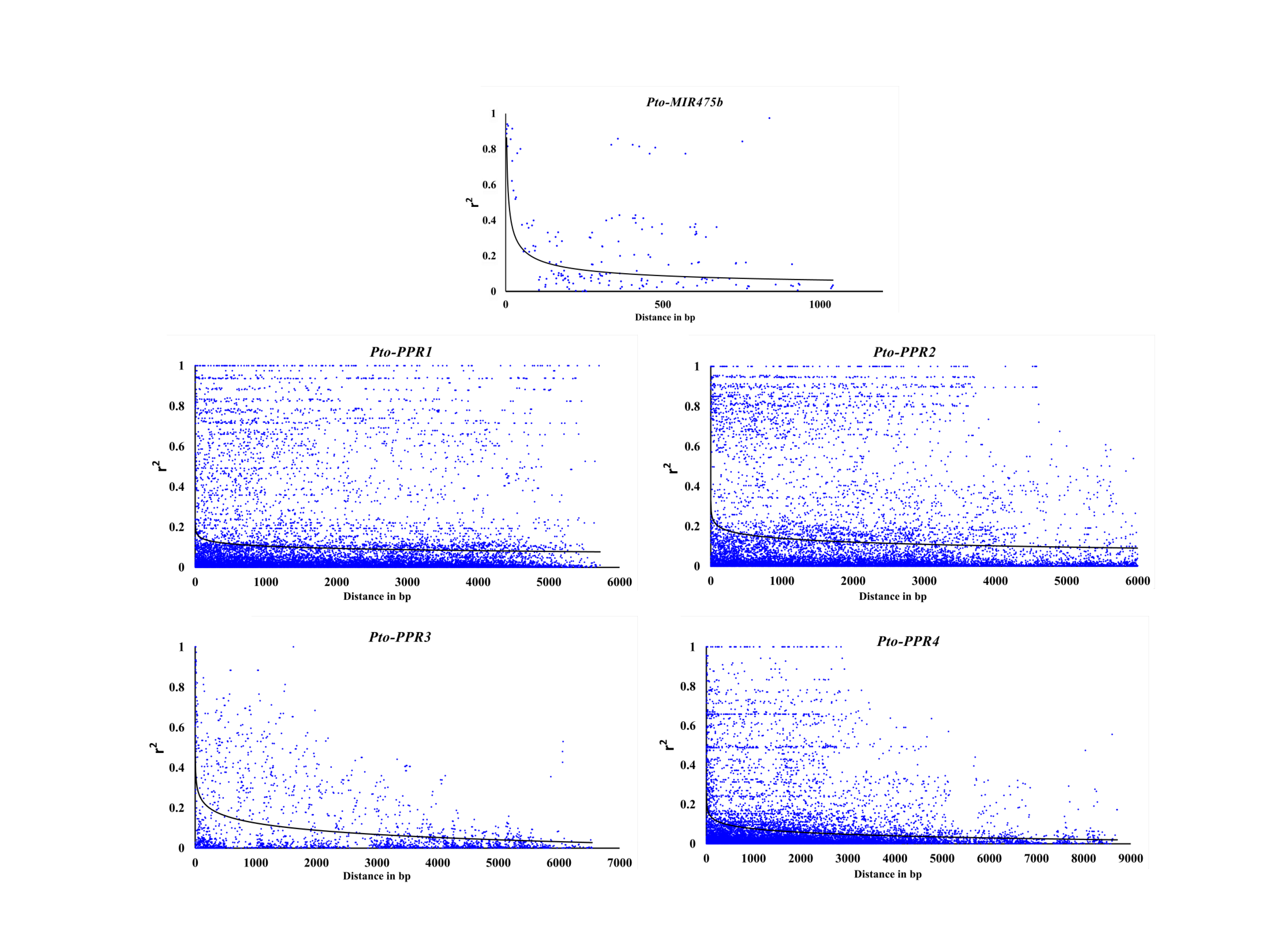

Supplement: FIGURE S1 — Decay of linkage disequilibrium of SNPs within Pto-MIR475b, Pto-PPR1, Pto-PPR2, Pto-PPR3, and Pto-PPR4. [file Image_1.TIF]

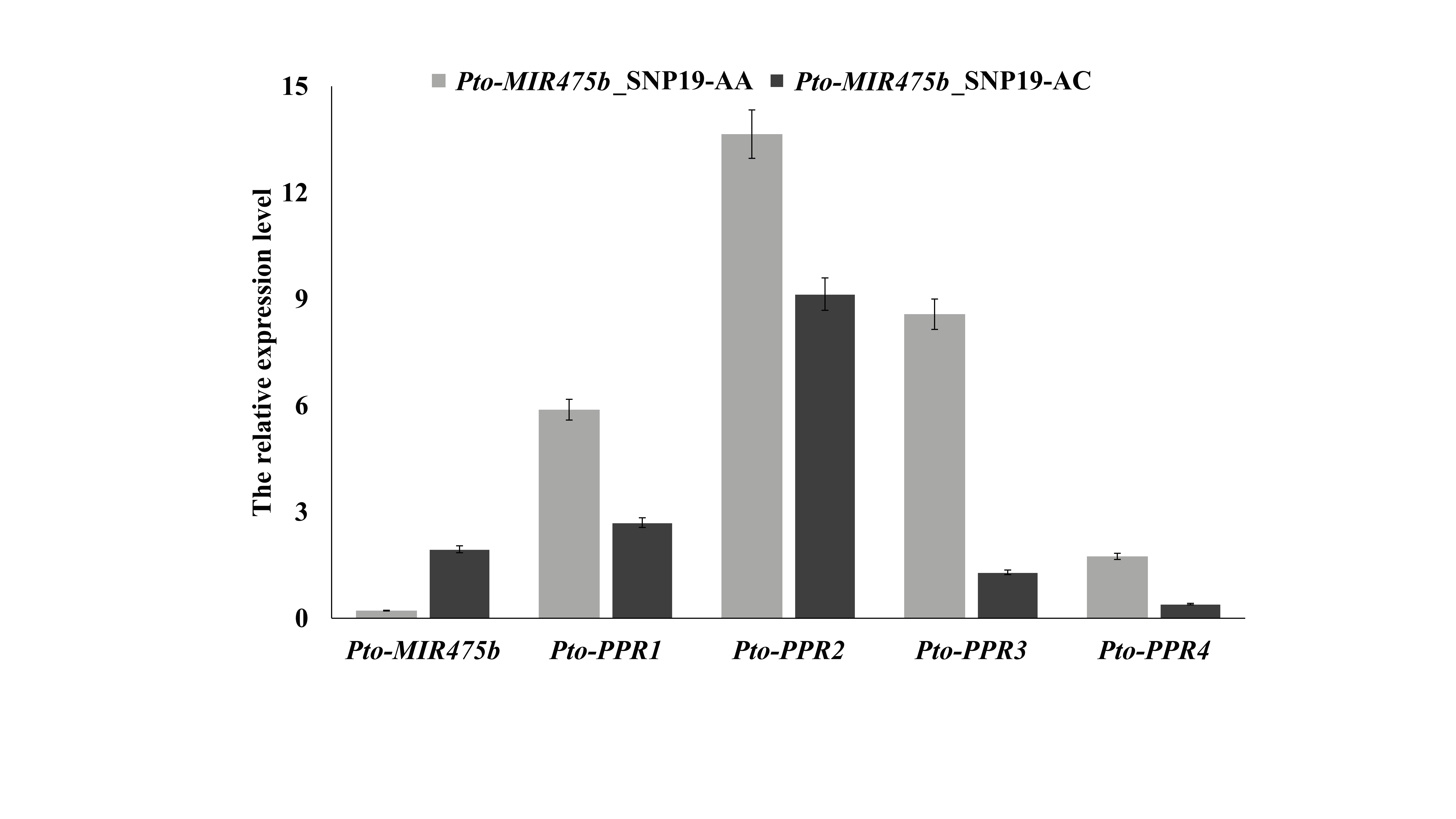

Supplement: FIGURE S2 — Expression levels of Pto-MIR475b and four target genes in the different genotypes of Pto-MIR475b_SNP19. [file Image_2.TIF]
