# Supplementary material for: Allelic Interactions among Pto-MIR475b and Its Four Target Genes Potentially Affect Growth and Wood Properties in Populus
Source: Front Plant Sci. 2017 Jun 21;8:1055. doi: 10.3389/fpls.2017.01055 (PMC5478899; doi:10.3389/fpls.2017.01055)
Supplement: Supplementary file 3 [file Table_1.DOCX]

| **Gene** | **Forward primer (5′→3′)** | **Reverse primer (5′→3′)** |
| --- | --- | --- |
| *Pto-MIR475b* | GGCTGGTTATTAGGGTGGATTG | GCGCAACTCTGGTTGACCTT |
| *Pto-PPR1* | TGAATCAAGGGCAGCACAAC | CCCCGCTTCTGCAACAAA |
| *Pto-PPR2* | TCCAGCACAAGGATGAATCAAG | TGCAACAAAACCTCTGTCTCTCA |
| *Pto-PPR3* | GTGCTCGATGGATGTGTTCCT | TCCCAAAATCCACCAACCA |
| *Pto-PPR4* | GGCAAGCTGTTCGCAACAA | GCTCTGTGATCATGTCCATTTCA |
| *Actin* | TTCATTTCACATCTTCCCCTTTT | GATCTCTGTGTGGGCGTCTGT |

**Table S1** The primers used for real-time PCR in this study.
