# Supplementary material for: Allelic Interactions among Pto-MIR475b and Its Four Target Genes Potentially Affect Growth and Wood Properties in Populus
Source: Front Plant Sci. 2017 Jun 21;8:1055. doi: 10.3389/fpls.2017.01055 (PMC5478899; doi:10.3389/fpls.2017.01055)
Supplement: Supplementary file 4 [file Table_2.DOCX]

**Table S2** Chromosomal location of the homologous *Pto-MIR475b* gene and four target genes in *P. trichocarpa*

| Gene | Homologous gene in  *P. trichocarpa* | Chromosome | Start position | End position |
| --- | --- | --- | --- | --- |
| *Pto-MIR475b* | *Ptr-MIR475b* | Chr08 | 14050496 | 14050632 |
| *Pto-PPR1* | Potri.006G242200 | Chr06 | 25089445 | 25091217 |
| *Pto-PPR2* | Potri.006G271200 | Chr06 | 27265727 | 27267859 |
| *Pto-PPR3* | Potri.011G057900 | Chr11 | 5112324 | 5115452 |
| *Pto-PPR4* | Potri.013G130600 | Chr13 | 14212198 | 14217006 |
