# Supplementary material for: Allelic Interactions among Pto-MIR475b and Its Four Target Genes Potentially Affect Growth and Wood Properties in Populus
Source: Front Plant Sci. 2017 Jun 21;8:1055. doi: 10.3389/fpls.2017.01055 (PMC5478899; doi:10.3389/fpls.2017.01055)
Supplement: Supplementary file 5 [file Table_3.DOCX]

**Table S3** The common SNPs in *Pto-MIR475b* and the four targets

| Gene | SNP locus | SNP position^a^ | Genotype | Region |
| --- | --- | --- | --- | --- |
| *Pto-MIR475b* |  |  |  |  |
|  | SNP1 | 2132 | A/G | Flanking |
|  | SNP2 | 2127 | C/T | Flanking |
|  | SNP3 | 2124 | G/T | Flanking |
|  | SNP4 | 1950 | A/G | Flanking |
|  | SNP5 | 1922 | A/G | Flanking |
|  | SNP6 | 1855 | A/G | Flanking |
|  | SNP7 | 1823 | A/G | Flanking |
|  | SNP8 | 1766 | A/T | Flanking |
|  | SNP9 | 1760 | A/G | Flanking |
|  | SNP10 | 1715 | A/G | Flanking |
|  | SNP11 | 1694 | C/T | Flanking |
|  | SNP12 | 1678 | A/G | Flanking |
|  | SNP13 | 1526 | C/T | Flanking |
|  | SNP14 | 1515 | C/T | Flanking |
|  | SNP15 | 1492 | C/T | Flanking |
|  | SNP16 | 1353 | A/G | Flanking |
|  | SNP17 | 1218 | A/C | Flanking |
|  | SNP18 | 1197 | A/G | Flanking |
|  | SNP19 | 1089 | A/C | Precursor |
| *Pto-PPR1* |  |  |  |  |
|  | SNP1 | 5759 | A/T | Down-stream |
|  | SNP2 | 5723 | C/T | Down-stream |
|  | SNP3 | 5623 | C/T | Down-stream |
|  | SNP4 | 5581 | C/T | Down-stream |
|  | SNP5 | 5540 | C/G | Down-stream |
|  | SNP6 | 5507 | A/T | Down-stream |
|  | SNP7 | 5430 | C/T | Down-stream |
|  | SNP8 | 5422 | C/T | Down-stream |
|  | SNP9 | 5419 | G/T | Down-stream |
|  | SNP10 | 5344 | C/T | Down-stream |
|  | SNP11 | 5305 | A/T | Down-stream |
|  | SNP12 | 5283 | A/C | Down-stream |
|  | SNP13 | 5160 | A/T | Down-stream |
|  | SNP14 | 5147 | A/T | Down-stream |
|  | SNP15 | 5138 | A/G | Down-stream |
|  | SNP16 | 5128 | A/T | Down-stream |
|  | SNP17 | 5075 | G/T | Down-stream |
|  | SNP18 | 4896 | C/G | Down-stream |
|  | SNP19 | 4795 | A/T | Down-stream |
|  | SNP20 | 4782 | C/G | Down-stream |
|  | SNP21 | 4750 | A/T | Down-stream |
|  | SNP22 | 4721 | G/T | Down-stream |
|  | SNP23 | 4720 | C/T | Down-stream |
|  | SNP24 | 4703 | C/G | Down-stream |
|  | SNP25 | 4687 | C/T | Down-stream |
|  | SNP26 | 4683 | C/T | Down-stream |
|  | SNP27 | 4679 | G/T | Down-stream |
|  | SNP28 | 4668 | A/G | Down-stream |
|  | SNP29 | 4620 | G/T | Down-stream |
|  | SNP30 | 4594 | A/T | Down-stream |
|  | SNP31 | 4586 | C/T | Down-stream |
|  | SNP32 | 4557 | C/T | Down-stream |
|  | SNP33 | 4546 | C/G | Down-stream |
|  | SNP34 | 4526 | A/C | Down-stream |
|  | SNP35 | 4508 | A/G | Down-stream |
|  | SNP36 | 4499 | A/G | Down-stream |
|  | SNP37 | 4471 | C/T | Down-stream |
|  | SNP38 | 4449 | A/C | Down-stream |
|  | SNP39 | 4409 | A/T | Down-stream |
|  | SNP40 | 4342 | A/G | Down-stream |
|  | SNP41 | 4257 | C/G | Down-stream |
|  | SNP42 | 4252 | C/G | Down-stream |
|  | SNP43 | 4211 | G/T | Down-stream |
|  | SNP44 | 4179 | A/T | Down-stream |
|  | SNP45 | 4163 | G/T | Down-stream |
|  | SNP46 | 4129 | G/T | Down-stream |
|  | SNP47 | 4127 | A/G | Down-stream |
|  | SNP48 | 4112 | A/G | Down-stream |
|  | SNP49 | 4056 | C/T | Down-stream |
|  | SNP50 | 4040 | A/G | Down-stream |
|  | SNP51 | 4022 | A/G | Down-stream |
|  | SNP52 | 3976 | C/G | Down-stream |
|  | SNP53 | 3958 | A/G | Down-stream |
|  | SNP54 | 3948 | C/T | Down-stream |
|  | SNP55 | 3930 | A/G | Down-stream |
|  | SNP56 | 3928 | A/C | Down-stream |
|  | SNP57 | 3913 | G/T | Down-stream |
|  | SNP58 | 3895 | A/G | Down-stream |
|  | SNP59 | 3863 | C/T | Down-stream |
|  | SNP60 | 3862 | A/G | Down-stream |
|  | SNP61 | 3802 | A/G | Down-stream |
|  | SNP62 | 3784 | C/G | Exon |
|  | SNP63 | 3781 | C/T | Exon |
|  | SNP64 | 3737 | C/T | Exon |
|  | SNP65 | 3672 | A/T | Exon |
|  | SNP66 | 3662 | C/T | Exon |
|  | SNP67 | 3599 | C/T | Exon |
|  | SNP68 | 3550 | A/G | Exon |
|  | SNP69 | 3479 | A/G | Exon |
|  | SNP70 | 3245 | A/G | Exon |
|  | SNP71 | 3180 | A/G | Exon |
|  | SNP72 | 3170 | A/G | Exon |
|  | SNP73 | 3164 | A/G | Exon |
|  | SNP74 | 3114 | A/G | Exon |
|  | SNP75 | 3059 | A/G | Exon |
|  | SNP76 | 3040 | C/T | Exon |
|  | SNP77 | 3024 | G/T | Exon |
|  | SNP78 | 2911 | C/G | Exon |
|  | SNP79 | 2747 | G/T | Exon |
|  | SNP80 | 2396 | A/G | Exon |
|  | SNP81 | 2285 | A/G | Exon |
|  | SNP82 | 2261 | G/T | Exon |
|  | SNP83 | 2229 | C/T | Exon |
|  | SNP84 | 2227 | C/T | Exon |
|  | SNP85 | 2219 | A/G | Exon |
|  | SNP86 | 2218 | G/T | Exon |
|  | SNP87 | 2097 | A/G | Exon |
|  | SNP88 | 1995 | A/C | Up-stream |
|  | SNP89 | 1994 | A/C | Up-stream |
|  | SNP90 | 1990 | G/T | Up-stream |
|  | SNP91 | 1989 | G/T | Up-stream |
|  | SNP92 | 1979 | G/T | Up-stream |
|  | SNP93 | 1893 | A/G | Up-stream |
|  | SNP94 | 1862 | G/T | Up-stream |
|  | SNP95 | 1854 | C/T | Up-stream |
|  | SNP96 | 1843 | A/G | Up-stream |
|  | SNP97 | 1827 | A/G | Up-stream |
|  | SNP98 | 1826 | C/G | Up-stream |
|  | SNP99 | 1757 | G/T | Up-stream |
|  | SNP100 | 1746 | C/T | Up-stream |
|  | SNP101 | 1740 | C/T | Up-stream |
|  | SNP102 | 1714 | A/T | Up-stream |
|  | SNP103 | 1692 | G/T | Up-stream |
|  | SNP104 | 1651 | C/T | Up-stream |
|  | SNP105 | 1645 | C/T | Up-stream |
|  | SNP106 | 1624 | A/T | Up-stream |
|  | SNP107 | 1561 | G/T | Up-stream |
|  | SNP108 | 1517 | C/T | Up-stream |
|  | SNP109 | 1509 | A/C | Up-stream |
|  | SNP110 | 1508 | A/T | Up-stream |
|  | SNP111 | 1496 | A/G | Up-stream |
|  | SNP112 | 1449 | A/G | Up-stream |
|  | SNP113 | 1422 | C/G | Up-stream |
|  | SNP114 | 1418 | C/T | Up-stream |
|  | SNP115 | 1408 | A/T | Up-stream |
|  | SNP116 | 1403 | A/T | Up-stream |
|  | SNP117 | 1399 | A/G | Up-stream |
|  | SNP118 | 1389 | A/C | Up-stream |
|  | SNP119 | 1386 | A/G | Up-stream |
|  | SNP120 | 1320 | A/T | Up-stream |
|  | SNP121 | 1306 | G/T | Up-stream |
|  | SNP122 | 1302 | A/C | Up-stream |
|  | SNP123 | 1293 | G/T | Up-stream |
|  | SNP124 | 1281 | C/T | Up-stream |
|  | SNP125 | 1274 | A/G | Up-stream |
|  | SNP126 | 1253 | C/T | Up-stream |
|  | SNP127 | 1216 | C/T | Up-stream |
|  | SNP128 | 1049 | C/T | Up-stream |
|  | SNP129 | 1013 | A/G | Up-stream |
|  | SNP130 | 996 | A/C | Up-stream |
|  | SNP131 | 988 | A/G | Up-stream |
|  | SNP132 | 964 | C/T | Up-stream |
|  | SNP133 | 943 | C/T | Up-stream |
|  | SNP134 | 852 | A/T | Up-stream |
|  | SNP135 | 847 | A/C | Up-stream |
|  | SNP136 | 828 | A/C | Up-stream |
|  | SNP137 | 785 | C/T | Up-stream |
|  | SNP138 | 775 | C/T | Up-stream |
|  | SNP139 | 773 | C/T | Up-stream |
|  | SNP140 | 750 | C/T | Up-stream |
|  | SNP141 | 742 | C/T | Up-stream |
|  | SNP142 | 722 | A/T | Up-stream |
|  | SNP143 | 687 | A/T | Up-stream |
|  | SNP144 | 678 | C/T | Up-stream |
|  | SNP145 | 632 | C/T | Up-stream |
|  | SNP146 | 631 | A/T | Up-stream |
|  | SNP147 | 578 | A/T | Up-stream |
|  | SNP148 | 486 | A/G | Up-stream |
|  | SNP149 | 478 | A/G | Up-stream |
|  | SNP150 | 419 | A/G | Up-stream |
|  | SNP151 | 355 | A/C | Up-stream |
|  | SNP152 | 346 | A/C | Up-stream |
|  | SNP153 | 325 | C/G | Up-stream |
|  | SNP154 | 120 | A/C | Up-stream |
|  | SNP155 | 119 | A/G | Up-stream |
|  | SNP156 | 107 | A/G | Up-stream |
|  | SNP157 | 48 | A/G | Up-stream |
| *Pto-PPR2* |  |  |  |  |
|  | SNP1 | 6116 | A/G | Down-stream |
|  | SNP2 | 6092 | A/C | Down-stream |
|  | SNP3 | 6086 | C/G | Down-stream |
|  | SNP4 | 6080 | C/T | Down-stream |
|  | SNP5 | 6060 | C/T | Down-stream |
|  | SNP6 | 6045 | A/C | Down-stream |
|  | SNP7 | 6004 | A/G | Down-stream |
|  | SNP8 | 5999 | A/C | Down-stream |
|  | SNP9 | 5969 | A/G | Down-stream |
|  | SNP10 | 5961 | A/T | Down-stream |
|  | SNP11 | 5917 | A/G | Down-stream |
|  | SNP12 | 5864 | A/G | Down-stream |
|  | SNP13 | 5801 | C/T | Down-stream |
|  | SNP14 | 5783 | A/G | Down-stream |
|  | SNP15 | 5686 | A/G | Down-stream |
|  | SNP16 | 5654 | C/T | Down-stream |
|  | SNP17 | 5649 | A/T | Down-stream |
|  | SNP18 | 5646 | C/T | Down-stream |
|  | SNP19 | 5632 | A/T | Down-stream |
|  | SNP20 | 5604 | G/T | Down-stream |
|  | SNP21 | 5581 | C/T | Down-stream |
|  | SNP22 | 5470 | A/G | Down-stream |
|  | SNP23 | 5465 | A/G | Down-stream |
|  | SNP24 | 5441 | G/T | Down-stream |
|  | SNP25 | 5431 | A/T | Down-stream |
|  | SNP26 | 5363 | A/G | Down-stream |
|  | SNP27 | 5351 | C/T | Down-stream |
|  | SNP28 | 5347 | C/T | Down-stream |
|  | SNP29 | 5343 | C/T | Down-stream |
|  | SNP30 | 5320 | C/G | Down-stream |
|  | SNP31 | 5284 | C/T | Down-stream |
|  | SNP32 | 5218 | A/T | Down-stream |
|  | SNP33 | 5122 | A/G | Down-stream |
|  | SNP34 | 5089 | A/T | Down-stream |
|  | SNP35 | 5050 | C/T | Down-stream |
|  | SNP36 | 5030 | A/T | Down-stream |
|  | SNP37 | 4965 | C/T | Down-stream |
|  | SNP38 | 4942 | A/G | Down-stream |
|  | SNP39 | 4938 | A/C | Down-stream |
|  | SNP40 | 4931 | C/T | Down-stream |
|  | SNP41 | 4800 | A/G | Down-stream |
|  | SNP42 | 4762 | A/G | Down-stream |
|  | SNP43 | 4745 | A/G | Down-stream |
|  | SNP44 | 4699 | A/G | Down-stream |
|  | SNP45 | 4671 | A/G | Down-stream |
|  | SNP46 | 4632 | G/T | Down-stream |
|  | SNP47 | 4564 | A/G | Down-stream |
|  | SNP48 | 4522 | A/C | Down-stream |
|  | SNP49 | 4458 | A/C | Down-stream |
|  | SNP50 | 4298 | A/G | Down-stream |
|  | SNP51 | 4295 | A/C | Down-stream |
|  | SNP52 | 4270 | A/T | Down-stream |
|  | SNP53 | 4267 | A/G | Down-stream |
|  | SNP54 | 4231 | A/T | Down-stream |
|  | SNP55 | 4209 | A/G | Down-stream |
|  | SNP56 | 4159 | A/C | Down-stream |
|  | SNP57 | 4047 | A/C | 3'UTR |
|  | SNP58 | 4038 | G/T | 3'UTR |
|  | SNP59 | 4004 | A/G | 3'UTR |
|  | SNP60 | 3996 | C/T | 3'UTR |
|  | SNP61 | 3965 | C/T | Exon |
|  | SNP62 | 3846 | C/G | Exon |
|  | SNP63 | 3833 | C/T | Exon |
|  | SNP64 | 3824 | C/T | Exon |
|  | SNP65 | 3697 | C/T | Exon |
|  | SNP66 | 3489 | G/T | Exon |
|  | SNP67 | 3418 | C/T | Exon |
|  | SNP68 | 3400 | C/G | Exon |
|  | SNP69 | 3393 | C/T | Exon |
|  | SNP70 | 3322 | C/T | Exon |
|  | SNP71 | 3318 | A/G | Exon |
|  | SNP72 | 3255 | C/T | Exon |
|  | SNP73 | 3245 | C/T | Exon |
|  | SNP74 | 3203 | A/T | Exon |
|  | SNP75 | 3197 | A/T | Exon |
|  | SNP76 | 3196 | A/T | Exon |
|  | SNP77 | 3195 | A/C | Exon |
|  | SNP78 | 3147 | C/T | Exon |
|  | SNP79 | 3138 | A/C | Exon |
|  | SNP80 | 3096 | A/C | Exon |
|  | SNP81 | 3093 | C/T | Exon |
|  | SNP82 | 3039 | A/G | Exon |
|  | SNP83 | 3015 | A/T | Exon |
|  | SNP84 | 3008 | C/T | Exon |
|  | SNP85 | 2993 | G/T | Exon |
|  | SNP86 | 2897 | A/C | Exon |
|  | SNP87 | 2860 | C/T | Exon |
|  | SNP88 | 2828 | G/T | Exon |
|  | SNP89 | 2819 | A/G | Exon |
|  | SNP90 | 2803 | C/T | Exon |
|  | SNP91 | 2790 | A/C | Exon |
|  | SNP92 | 2764 | A/G | 5'UTR |
|  | SNP93 | 2752 | C/T | 5'UTR |
|  | SNP94 | 2694 | A/T | 5'UTR |
|  | SNP95 | 2672 | A/C | 5'UTR |
|  | SNP96 | 2620 | A/C | Intron |
|  | SNP97 | 2615 | C/T | Intron |
|  | SNP98 | 2610 | G/T | Intron |
|  | SNP99 | 2607 | C/G | Intron |
|  | SNP100 | 2606 | C/T | Intron |
|  | SNP101 | 2596 | A/G | Intron |
|  | SNP102 | 2581 | A/G | Intron |
|  | SNP103 | 2578 | A/T | Intron |
|  | SNP104 | 2577 | C/T | Intron |
|  | SNP105 | 2565 | C/T | Intron |
|  | SNP106 | 2510 | C/T | Intron |
|  | SNP107 | 2504 | C/T | Intron |
|  | SNP108 | 2503 | A/G | Intron |
|  | SNP109 | 2487 | A/T | Intron |
|  | SNP110 | 2468 | G/T | Intron |
|  | SNP111 | 2408 | A/G | Intron |
|  | SNP112 | 2392 | G/T | Intron |
|  | SNP113 | 2390 | A/G | Intron |
|  | SNP114 | 2279 | A/T | 5'UTR |
|  | SNP115 | 2227 | A/G | 5'UTR |
|  | SNP116 | 2200 | C/T | 5'UTR |
|  | SNP117 | 2179 | A/G | 5'UTR |
|  | SNP118 | 2168 | G/T | 5'UTR |
|  | SNP119 | 2103 | A/G | 5'UTR |
|  | SNP120 | 2098 | C/G | 5'UTR |
|  | SNP121 | 2063 | G/T | 5'UTR |
|  | SNP122 | 2040 | A/G | 5'UTR |
|  | SNP123 | 1964 | C/T | Up-stream |
|  | SNP124 | 1959 | G/T | Up-stream |
|  | SNP125 | 1889 | G/T | Up-stream |
|  | SNP126 | 266 | C/T | Up-stream |
|  | SNP127 | 240 | C/T | Up-stream |
|  | SNP128 | 195 | A/G | Up-stream |
|  | SNP129 | 166 | C/T | Up-stream |
|  | SNP130 | 163 | A/G | Up-stream |
|  | SNP131 | 155 | G/T | Up-stream |
|  | SNP132 | 105 | C/T | Up-stream |
|  | SNP133 | 98 | A/T | Up-stream |
|  | SNP134 | 86 | C/T | Up-stream |
|  | SNP135 | 82 | A/G | Up-stream |
|  | SNP136 | 79 | A/G | Up-stream |
|  | SNP137 | 77 | A/G | Up-stream |
|  | SNP138 | 35 | G/T | Up-stream |
|  | SNP139 | 34 | C/G | Up-stream |
|  | SNP140 | 32 | A/T | Up-stream |
|  | SNP141 | 30 | G/T | Up-stream |
|  | SNP142 | 27 | A/T | Up-stream |
|  | SNP143 | 24 | C/T | Up-stream |
|  | SNP144 | 6 | A/T | Up-stream |
| *Pto-PPR3* |  |  |  |  |
|  | SNP1 | 343 | A/T | Up-stream |
|  | SNP2 | 344 | G/T | Up-stream |
|  | SNP3 | 349 | A/T | Up-stream |
|  | SNP4 | 351 | A/T | Up-stream |
|  | SNP5 | 355 | A/T | Up-stream |
|  | SNP6 | 357 | A/T | Up-stream |
|  | SNP7 | 365 | A/G | Up-stream |
|  | SNP8 | 391 | C/T | Up-stream |
|  | SNP9 | 405 | C/T | Up-stream |
|  | SNP10 | 412 | A/G | Up-stream |
|  | SNP11 | 421 | A/T | Up-stream |
|  | SNP12 | 430 | C/T | Up-stream |
|  | SNP13 | 486 | G/T | Up-stream |
|  | SNP14 | 559 | C/G | Up-stream |
|  | SNP15 | 566 | A/C | Up-stream |
|  | SNP16 | 617 | A/G | Up-stream |
|  | SNP17 | 633 | G/T | Up-stream |
|  | SNP18 | 656 | A/T | Up-stream |
|  | SNP19 | 668 | A/G | Up-stream |
|  | SNP20 | 669 | A/C | Up-stream |
|  | SNP21 | 680 | A/C | Up-stream |
|  | SNP22 | 715 | A/G | Up-stream |
|  | SNP23 | 741 | A/T | Up-stream |
|  | SNP24 | 802 | A/G | Up-stream |
|  | SNP25 | 804 | C/T | Up-stream |
|  | SNP26 | 815 | C/T | Up-stream |
|  | SNP27 | 821 | A/T | Up-stream |
|  | SNP28 | 829 | A/G | Up-stream |
|  | SNP29 | 830 | A/G | Up-stream |
|  | SNP30 | 836 | A/C | Up-stream |
|  | SNP31 | 1855 | C/T | Up-stream |
|  | SNP32 | 1909 | A/C | Up-stream |
|  | SNP33 | 1918 | A/C | Up-stream |
|  | SNP34 | 1932 | C/T | Up-stream |
|  | SNP35 | 2649 | C/T | Exon |
|  | SNP36 | 2748 | A/G | Exon |
|  | SNP37 | 2867 | A/G | Exon |
|  | SNP38 | 3737 | C/T | 3'UTR |
|  | SNP39 | 3788 | C/G | 3'UTR |
|  | SNP40 | 3825 | C/T | Intron |
|  | SNP41 | 3826 | A/C | Intron |
|  | SNP42 | 3874 | G/T | Intron |
|  | SNP43 | 3985 | G/T | Intron |
|  | SNP44 | 4003 | A/G | Intron |
|  | SNP45 | 4014 | A/G | Intron |
|  | SNP46 | 4359 | C/T | Intron |
|  | SNP47 | 4496 | C/T | Intron |
|  | SNP48 | 4525 | C/T | Intron |
|  | SNP49 | 4570 | A/G | Intron |
|  | SNP50 | 4909 | A/G | Intron |
|  | SNP51 | 4927 | C/T | Intron |
|  | SNP52 | 4953 | G/T | Intron |
|  | SNP53 | 4987 | C/T | Intron |
|  | SNP54 | 5279 | A/G | Down-stream |
|  | SNP55 | 5289 | A/G | Down-stream |
|  | SNP56 | 5391 | C/T | Down-stream |
|  | SNP57 | 5401 | C/G | Down-stream |
|  | SNP58 | 5673 | G/T | Down-stream |
|  | SNP59 | 5679 | A/T | Down-stream |
|  | SNP60 | 5783 | A/T | Down-stream |
|  | SNP61 | 5800 | A/T | Down-stream |
|  | SNP62 | 5828 | A/C | Down-stream |
|  | SNP63 | 5980 | A/T | Down-stream |
|  | SNP64 | 6164 | C/T | Down-stream |
|  | SNP65 | 6185 | C/T | Down-stream |
|  | SNP66 | 6195 | G/T | Down-stream |
|  | SNP67 | 6535 | A/G | Down-stream |
|  | SNP68 | 6889 | A/G | Down-stream |
| *Pto-PPR4* |  |  |  |  |
|  | SNP1 | 8825 | G/T | Up-stream |
|  | SNP2 | 8824 | C/T | Up-stream |
|  | SNP3 | 8791 | A/G | Up-stream |
|  | SNP4 | 8736 | A/G | Up-stream |
|  | SNP5 | 8727 | C/G | Up-stream |
|  | SNP6 | 8726 | C/T | Up-stream |
|  | SNP7 | 8722 | C/G | Up-stream |
|  | SNP8 | 8720 | A/G | Up-stream |
|  | SNP9 | 8698 | A/T | Up-stream |
|  | SNP10 | 8644 | A/C | Up-stream |
|  | SNP11 | 8609 | A/G | Up-stream |
|  | SNP12 | 8594 | A/G | Up-stream |
|  | SNP13 | 8203 | A/T | Up-stream |
|  | SNP14 | 8158 | A/T | Up-stream |
|  | SNP15 | 8139 | A/G | Up-stream |
|  | SNP16 | 8104 | A/G | Up-stream |
|  | SNP17 | 8084 | C/T | Up-stream |
|  | SNP18 | 8057 | A/G | Up-stream |
|  | SNP19 | 8009 | A/G | Up-stream |
|  | SNP20 | 7991 | C/T | Up-stream |
|  | SNP21 | 7963 | C/T | Up-stream |
|  | SNP22 | 7961 | C/T | Up-stream |
|  | SNP23 | 7936 | A/G | Up-stream |
|  | SNP24 | 7869 | A/T | Up-stream |
|  | SNP25 | 7552 | A/G | Up-stream |
|  | SNP26 | 7551 | A/T | Up-stream |
|  | SNP27 | 7519 | A/T | Up-stream |
|  | SNP28 | 7501 | C/T | Up-stream |
|  | SNP29 | 7333 | C/T | Up-stream |
|  | SNP30 | 7313 | C/T | Up-stream |
|  | SNP31 | 7308 | A/T | Up-stream |
|  | SNP32 | 7305 | C/T | Up-stream |
|  | SNP33 | 7301 | G/T | Up-stream |
|  | SNP34 | 7288 | A/G | Up-stream |
|  | SNP35 | 7210 | A/G | Up-stream |
|  | SNP36 | 7201 | A/C | Up-stream |
|  | SNP37 | 7179 | C/T | Up-stream |
|  | SNP38 | 7171 | C/T | Up-stream |
|  | SNP39 | 7124 | G/T | Up-stream |
|  | SNP40 | 7015 | A/G | Up-stream |
|  | SNP41 | 6995 | C/T | Up-stream |
|  | SNP42 | 6986 | G/T | Up-stream |
|  | SNP43 | 6967 | A/T | Up-stream |
|  | SNP44 | 6961 | A/T | Up-stream |
|  | SNP45 | 6946 | A/G | Up-stream |
|  | SNP46 | 6926 | C/G | Up-stream |
|  | SNP47 | 6891 | A/G | Up-stream |
|  | SNP48 | 6862 | C/T | Up-stream |
|  | SNP49 | 6836 | A/G | 3'UTR |
|  | SNP50 | 6831 | A/C | 3'UTR |
|  | SNP51 | 6817 | A/T | 3'UTR |
|  | SNP52 | 6763 | A/G | 3'UTR |
|  | SNP53 | 6730 | C/T | 3'UTR |
|  | SNP54 | 6728 | G/T | 3'UTR |
|  | SNP55 | 6726 | A/G | 3'UTR |
|  | SNP56 | 6719 | C/T | 3'UTR |
|  | SNP57 | 6718 | G/T | 3'UTR |
|  | SNP58 | 6716 | A/C | 3'UTR |
|  | SNP59 | 6667 | C/T | 3'UTR |
|  | SNP60 | 6604 | A/G | 3'UTR |
|  | SNP61 | 6535 | C/T | 3'UTR |
|  | SNP62 | 6459 | C/T | Intron |
|  | SNP63 | 6413 | C/T | Intron |
|  | SNP64 | 6358 | C/T | Intron |
|  | SNP65 | 6346 | A/G | Intron |
|  | SNP66 | 6222 | C/T | Intron |
|  | SNP67 | 6219 | A/G | Intron |
|  | SNP68 | 6216 | A/G | Intron |
|  | SNP69 | 6199 | A/T | Intron |
|  | SNP70 | 6198 | A/T | Intron |
|  | SNP71 | 6192 | A/G | Intron |
|  | SNP72 | 6185 | A/G | Intron |
|  | SNP73 | 6096 | A/G | Intron |
|  | SNP74 | 5849 | A/G | 3'UTR |
|  | SNP75 | 5647 | C/T | Intron |
|  | SNP76 | 5569 | A/T | Intron |
|  | SNP77 | 5538 | A/C | Intron |
|  | SNP78 | 5532 | A/G | Intron |
|  | SNP79 | 5493 | A/C | Intron |
|  | SNP80 | 5436 | A/G | Intron |
|  | SNP81 | 5435 | A/C | Intron |
|  | SNP82 | 5430 | A/G | Intron |
|  | SNP83 | 5386 | C/T | Intron |
|  | SNP84 | 5343 | C/T | Intron |
|  | SNP85 | 5255 | C/G | Intron |
|  | SNP86 | 5251 | A/T | Intron |
|  | SNP87 | 5247 | A/T | Intron |
|  | SNP88 | 5224 | A/T | Intron |
|  | SNP89 | 5208 | A/C | Intron |
|  | SNP90 | 5207 | A/T | Intron |
|  | SNP91 | 5194 | A/C | Intron |
|  | SNP92 | 5193 | A/T | Intron |
|  | SNP93 | 5188 | C/T | Intron |
|  | SNP94 | 5172 | C/T | Intron |
|  | SNP95 | 5169 | C/T | Intron |
|  | SNP96 | 5139 | G/T | Intron |
|  | SNP97 | 5085 | C/T | Intron |
|  | SNP98 | 5073 | G/T | Intron |
|  | SNP99 | 5072 | A/T | Intron |
|  | SNP100 | 5059 | A/G | Intron |
|  | SNP101 | 5042 | C/T | Intron |
|  | SNP102 | 5041 | A/G | Intron |
|  | SNP103 | 5028 | A/G | Intron |
|  | SNP104 | 4839 | A/G | Intron |
|  | SNP105 | 4838 | A/G | Intron |
|  | SNP106 | 4825 | G/T | Intron |
|  | SNP107 | 4800 | A/T | Intron |
|  | SNP108 | 4740 | A/T | Intron |
|  | SNP109 | 4737 | A/T | Intron |
|  | SNP110 | 4710 | A/T | Intron |
|  | SNP111 | 4709 | C/T | Intron |
|  | SNP112 | 4694 | A/C | Intron |
|  | SNP113 | 4570 | A/G | Intron |
|  | SNP114 | 4539 | A/T | Intron |
|  | SNP115 | 4501 | A/T | Intron |
|  | SNP116 | 4394 | C/T | Intron |
|  | SNP117 | 4288 | A/G | Intron |
|  | SNP118 | 4109 | G/T | Intron |
|  | SNP119 | 4015 | A/T | Intron |
|  | SNP120 | 4014 | C/G | Intron |
|  | SNP121 | 3901 | A/G | Intron |
|  | SNP122 | 3876 | C/T | Intron |
|  | SNP123 | 3725 | A/G | Intron |
|  | SNP124 | 3709 | A/G | Intron |
|  | SNP125 | 3653 | A/T | Intron |
|  | SNP126 | 3385 | C/T | 3'UTR |
|  | SNP127 | 3371 | C/T | 3'UTR |
|  | SNP128 | 3204 | A/G | Intron |
|  | SNP129 | 3130 | G/T | Intron |
|  | SNP130 | 486 | A/G | Down-stream |
|  | SNP131 | 387 | A/T | Down-stream |
|  | SNP132 | 344 | G/T | Down-stream |
|  | SNP133 | 119 | C/T | Down-stream |

^a^SNP position: The SNP position in genes (bp).
