# Supplementary material for: Allelic Interactions among Pto-MIR475b and Its Four Target Genes Potentially Affect Growth and Wood Properties in Populus
Source: Front Plant Sci. 2017 Jun 21;8:1055. doi: 10.3389/fpls.2017.01055 (PMC5478899; doi:10.3389/fpls.2017.01055)
Supplement: Supplementary file 6 [file Table_4.DOCX]

**Table S4** Single-nucleotide polymorphisms of *Pto-MIR475b* and the four target genes

| Gene | Region | Length (bp) | No. of polymorphic sites | Frequency (bp-1) | Nucleotide diversity | |
| --- | --- | --- | --- | --- | --- | --- |
|  |  |  |  |  | π | θw |
| *Pto-MIR475b* |  |  |  |  |  |  |
|  | Pre-mature region | 135 | 1 | 1/135 | 0.00137 | 0.00137 |
|  | Total | 2135 | 19 | 1/112 | 0.00208 | 0.00208 |
| *Pto-PPR1* |  |  |  |  |  |  |
|  | Upstream | 2000 | 70 | 1/29 | 0.01199 | 0.00823 |
|  | Exon | 1794 | 26 | 1/69 | 0.00482 | 0.00341 |
|  | Downstream | 2000 | 61 | 1/33 | 0.01174 | 0.00717 |
|  | Synonymous | 402.04 | 11 | 1/37 | 0.00869 | 0.00643 |
|  | Non-synonymous | 1388.96 | 15 | 1/93 | 0.00371 | 0.00254 |
|  | Total silent^a^ | 4405.04 | 142 | 1/31 | 0.01157 | 0.00758 |
|  | Total^b^ | 5794 | 159 | 1/36 | 0.0097 | 0.00645 |
| *Pto-PPR2* |  |  |  |  |  |  |
|  | Upstream | 2000 | 22 | 1/91 | 0.0038 | 0.00259 |
|  | 5' UTR 1 | 320 | 9 | 1/36 | 0.01197 | 0.00661 |
|  | Intron1 | 317 | 18 | 1/18 | 0.02376 | 0.01335 |
|  | 3' UTR 2 | 143 | 5 | 1/29 | 0.01187 | 0.00816 |
|  | Exon | 1212 | 39 | 1/31 | 0.01217 | 0.00757 |
|  | 3' UTR | 138 | 5 | 1/28 | 0.01548 | 0.00852 |
|  | Downstream | 2000 | 58 | 1/34 | 0.01023 | 0.00682 |
|  | Synonymous | 271.03 | 11 | 1/25 | 0.015 | 0.00954 |
|  | Non-synonymous | 937.98 | 28 | 1/33 | 0.01139 | 0.00702 |
|  | Total silent^a^ | 5191.02 | 128 | 1/41 | 0.00912 | 0.0058 |
|  | Total^b^ | 6130 | 156 | 1/39 | 0.00946 | 0.00598 |
| *Pto-PPR3* |  |  |  |  |  |  |
|  | Upstream | 2000 | 35 | 1/57 | 0.00558 | 0.00411 |
|  | 5' UTR | 145 | 0 | - | 0 | 0 |
|  | Exon | 1437 | 3 | 1/479 | 0.00055 | 0.00049 |
|  | 3' UTR 1 | 217 | 2 | 1/109 | 0.00151 | 0.00217 |
|  | Intron | 1213 | 14 | 1/87 | 0.00297 | 0.00271 |
|  | 3' UTR 2 | 0 | 0 | - | 0 | 0 |
|  | Downstream | 2000 | 15 | 1/133 | 0.00141 | 0.00176 |
|  | Synonymous | 310.83 | 2 | 1/155 | 0.0021 | 0.00151 |
|  | Non-synonymous | 1123.17 | 1 | 1/1123 | 0.00013 | 0.00021 |
|  | Total silent^a^ | 6004.83 | 68 | 1/88 | 0.00309 | 0.00266 |
|  | Total^b^ | 7128 | 69 | 1/103 | 0.00262 | 0.00228 |
| *Pto-PPR4* |  |  |  |  |  |  |
|  | Upstream | 2000 | 7 | 1/286 | 0.00084 | 0.00082 |
|  | Exon 1 | 1110 | 0 | - | 0 | 0 |
|  | Intron 1 | 116 | 2 | 1/58 | 0.00414 | 0.00405 |
|  | Exon 2 | 45 | 0 | - | 0 | 0 |
|  | 3' UTR 1 | 190 | 2 | 1/95 | 0.00538 | 0.00247 |
|  | Intron 2 | 2386 | 51 | 1/47 | 0.00794 | 0.00503 |
|  | 3' UTR 2 | 215 | 1 | 1/215 | 0.00104 | 0.00109 |
|  | Intron 3 | 443 | 12 | 1/37 | 0.00929 | 0.00637 |
|  | 3' UTR 3 | 340 | 13 | 1/26 | 0.01298 | 0.00899 |
|  | Downstream | 2000 | 53 | 1/38 | 0.00838 | 0.00623 |
|  | Synonymous | 259.5 | 0 | - | 0 | 0 |
|  | Non-synonymous | 892.5 | 0 | - | 0 | 0 |
|  | Total silent^a^ | 7952.5 | 141 | 1/56 | 0.00599 | 0.00417 |
|  | Total^b^ | 8845 | 141 | 1/63 | 0.00538 | 0.00375 |
