# Supplementary material for: Allelic Interactions among Pto-MIR475b and Its Four Target Genes Potentially Affect Growth and Wood Properties in Populus
Source: Front Plant Sci. 2017 Jun 21;8:1055. doi: 10.3389/fpls.2017.01055 (PMC5478899; doi:10.3389/fpls.2017.01055)
Supplement: Supplementary file 7 [file Table_5.DOCX]

**Table S5** Detail of significant SNPs within *Pto-MIR475b* and four targets associated with tree growth and wood formation in a natural population of *P. tomentosa*

| Trait | Marker | Genotype | *P*-value | *Q-value* | *R^2^* (%) |
| --- | --- | --- | --- | --- | --- |
| CC (%) |  |  |  |  |  |
|  | Pto-PPR4_SNP107 | A/T | 0.0017 | 0.0896216 | 14.67 |
|  | Pto-PPR4_SNP130 | A/G | 7.13E-04 | 0.0599266 | 17.97 |
| DBH (cm) |  |  |  |  |  |
|  | Pto-PPR3_SNP27 | A/T | 0.0016 | 0.0869995 | 11.33 |
|  | Pto-PPR3_SNP28 | A/G | 1.16E-04 | 0.017981 | 20.11 |
|  | Pto-PPR3_SNP29 | A/G | 0.0013 | 0.0762782 | 11.75 |
|  | Pto-PPR4_SNP111 | C/T | 3.44E-42 | 5.10E-39 | 4.23 |
|  | Pto-PPR4_SNP73 | A/G | 0.0017 | 0.0896216 | 11.18 |
|  | Pto-PPR4_SNP86 | A/T | 1.18E-44 | 2.04E-41 | 4.19 |
|  | Pto-PPR1_SNP109 | A/C | 7.44E-46 | 1.55E-42 | 22.57 |
|  | Pto-PPR1_SNP114 | C/T | 4.67E-04 | 0.0500006 | 13.76 |
|  | Pto-PPR1_SNP115 | A/T | 1.72E-23 | 1.49E-20 | 2.21 |
|  | Pto-PPR1_SNP127 | C/T | 7.48E-04 | 0.0599266 | 12.85 |
|  | Pto-PPR1_SNP152 | A/C | 1.70E-04 | 0.0238587 | 19.83 |
|  | Pto-PPR1_SNP16 | A/T | 7.38E-26 | 7.66E-23 | 6.50 |
|  | Pto-PPR1_SNP41 | C/G | 3.11E-24 | 2.94E-21 | 7.50 |
|  | Pto-PPR1_SNP57 | G/T | 3.35E-04 | 0.039536 | 15.09 |
|  | Pto-PPR1_SNP67 | C/T | 7.36E-04 | 0.0599266 | 12.88 |
|  | Pto-PPR1_SNP90 | G/T | 4.96E-04 | 0.0525637 | 17.39 |
|  | Pto-PPR2_SNP105 | C/T | 0.0016 | 0.0869995 | 11.31 |
|  | Pto-PPR2_SNP106 | C/T | 7.29E-04 | 0.0599266 | 12.9 |
|  | Pto-PPR2_SNP108 | A/G | 5.43E-04 | 0.0537082 | 13.47 |
|  | Pto-PPR2_SNP115 | A/G | 5.16E-05 | 0.0086435 | 17.96 |
|  | Pto-PPR2_SNP116 | C/T | 5.16E-05 | 0.0086435 | 17.96 |
|  | Pto-PPR2_SNP120 | C/G | 4.36E-04 | 0.0471678 | 17.16 |
|  | Pto-PPR2_SNP121 | G/T | 5.16E-05 | 0.0086435 | 17.96 |
|  | Pto-PPR2_SNP122 | A/G | 5.16E-05 | 0.0086435 | 17.96 |
|  | Pto-PPR2_SNP123 | C/T | 2.11E-05 | 0.0086435 | 19.63 |
|  | Pto-PPR2_SNP141 | G/T | 2.80E-36 | 3.23E-33 | 6.00 |
|  | Pto-PPR2_SNP17 | A/T | 4.76E-62 | 4.94E-58 | 3.15 |
|  | Pto-PPR2_SNP18 | C/T | 5.16E-05 | 0.0086435 | 17.96 |
|  | Pto-PPR2_SNP20 | G/T | 7.29E-04 | 0.0599266 | 12.9 |
|  | Pto-PPR2_SNP30 | C/G | 1.40E-47 | 3.63E-44 | 1.50 |
|  | Pto-PPR2_SNP32 | A/T | 7.29E-04 | 0.0599266 | 12.9 |
|  | Pto-PPR2_SNP40 | C/T | 2.90E-04 | 0.0350211 | 17.97 |
|  | Pto-PPR2_SNP47 | A/G | 5.16E-05 | 0.0086435 | 17.96 |
|  | Pto-PPR2_SNP48 | A/C | 5.16E-05 | 0.0086435 | 17.96 |
|  | Pto-PPR2_SNP52 | A/T | 0.0013 | 0.0762782 | 11.98 |
|  | Pto-PPR2_SNP55 | A/G | 6.92E-05 | 0.0112294 | 20.78 |
|  | Pto-PPR2_SNP57 | A/C | 2.41E-04 | 0.0314163 | 18.35 |
|  | Pto-PPR2_SNP60 | C/T | 5.16E-05 | 0.0086435 | 17.96 |
|  | Pto-PPR2_SNP65 | C/T | 0.0015 | 0.0846649 | 11.47 |
|  | Pto-PPR2_SNP67 | C/T | 5.16E-05 | 0.0086435 | 17.96 |
|  | Pto-PPR2_SNP71 | A/G | 1.26E-04 | 0.0189649 | 19.61 |
|  | Pto-PPR2_SNP77 | A/C | 0.0015 | 0.0846649 | 11.54 |
|  | Pto-PPR2_SNP89 | A/G | 5.43E-04 | 0.0537082 | 13.47 |
| FW (µm) |  |  |  |  |  |
|  | Pto-MIR475b_SNP1 | A/G | 1.51E-04 | 0.0224031 | 16.25 |
|  | Pto-MIR475b_SNP2 | C/T | 5.64E-04 | 0.0537382 | 16.97 |
|  | Pto-PPR3_SNP17 | G/T | 0.0012 | 0.0728812 | 15.08 |
|  | Pto-PPR3_SNP65 | C/T | 7.66E-04 | 0.0599266 | 17.07 |
|  | Pto-PPR4_SNP117 | A/G | 0.0018 | 0.0934701 | 11.31 |
|  | Pto-PPR4_SNP63 | C/T | 9.14E-04 | 0.0599266 | 12.83 |
|  | Pto-PPR4_SNP65 | A/G | 3.66E-04 | 0.0422346 | 14.44 |
|  | Pto-PPR4_SNP79 | A/C | 0.0012 | 0.0728812 | 12.03 |
|  | Pto-PPR1_SNP121 | G/T | 9.23E-04 | 0.0599266 | 12.62 |
|  | Pto-PPR1_SNP20 | C/G | 0.0015 | 0.0846649 | 11.62 |
|  | Pto-PPR2_SNP81 | C/T | 4.00E-04 | 0.0446691 | 14.34 |
| H (m) |  |  |  |  |  |
|  | Pto-MIR475b_SNP19 | A/C | 3.46E-19 | 2.57E-16 | 2.62 |
|  | Pto-PPR3_SNP13 | G/T | 4.71E-10 | 3.06E-07 | 6.4 |
|  | Pto-PPR4_SNP18 | A/G | 2.72E-04 | 0.0340346 | 1.72 |
|  | Pto-PPR4_SNP44 | A/T | 7.89E-10 | 4.55E-07 | 2.13 |
|  | Pto-PPR4_SNP60 | A/G | 3.47E-12 | 2.40E-09 | 5.3 |
|  | Pto-PPR1_SNP12 | A/C | 5.07E-10 | 3.10E-07 | 3.35 |
|  | Pto-PPR1_SNP135 | A/C | 1.73E-07 | 8.17E-05 | 2.13 |
|  | Pto-PPR1_SNP98 | C/G | 1.07E-09 | 5.85E-07 | 2.03 |
|  | Pto-PPR2_SNP54 | A/T | 2.66E-05 | 0.0086435 | 5.2 |
| HC (%) |  |  |  |  |  |
|  | Pto-PPR1_SNP97 | A/G | 4.15E-04 | 0.0458512 | 16.45 |
| HEC (%) |  |  |  |  |  |
|  | Pto-PPR4_SNP89 | A/C | 0.0019 | 0.0948681 | 11.17 |
| LC (%) |  |  |  |  |  |
|  | Pto-PPR4_SNP21 | C/T | 0.0019 | 0.0948681 | 11.22 |
|  | Pto-PPR4_SNP24 | A/T | 0.0016 | 0.0869995 | 11.71 |
| MFA (º) |  |  |  |  |  |
|  | Pto-PPR4_SNP15 | A/G | 0.0011 | 0.06882 | 15.85 |
|  | Pto-PPR1_SNP101 | C/T | 9.72E-04 | 0.0623134 | 15.93 |
|  | Pto-PPR2_SNP112 | G/T | 0.0019 | 0.0948681 | 11.18 |
|  | Pto-PPR2_SNP113 | A/G | 0.0019 | 0.0948681 | 11.18 |
| V (m^3^) |  |  |  |  |  |
|  | Pto-PPR3_SNP28 | A/G | 9.20E-04 | 0.0599266 | 15.67 |
|  | Pto-PPR4_SNP112 | A/C | 5.60E-04 | 0.0537382 | 13.16 |
|  | Pto-PPR4_SNP115 | A/T | 5.60E-04 | 0.0537382 | 13.16 |
|  | Pto-PPR4_SNP49 | A/G | 2.23E-04 | 0.0300777 | 15.05 |
|  | Pto-PPR4_SNP56 | C/T | 3.83E-04 | 0.0437107 | 14.08 |
|  | Pto-PPR4_SNP74 | A/G | 3.44E-04 | 0.040142 | 14.09 |
|  | Pto-PPR1_SNP22 | G/T | 1.58E-04 | 0.0229348 | 18.83 |
|  | Pto-PPR2_SNP115 | A/G | 9.29E-04 | 0.0599266 | 12.19 |
|  | Pto-PPR2_SNP116 | C/T | 9.29E-04 | 0.0599266 | 12.19 |
|  | Pto-PPR2_SNP121 | G/T | 9.29E-04 | 0.0599266 | 12.19 |
|  | Pto-PPR2_SNP122 | A/G | 9.29E-04 | 0.0599266 | 12.19 |
|  | Pto-PPR2_SNP123 | C/T | 6.17E-04 | 0.0572133 | 12.98 |
|  | Pto-PPR2_SNP18 | C/T | 9.29E-04 | 0.0599266 | 12.19 |
|  | Pto-PPR2_SNP47 | A/G | 9.29E-04 | 0.0599266 | 12.19 |
|  | Pto-PPR2_SNP48 | A/C | 9.29E-04 | 0.0599266 | 12.19 |
|  | Pto-PPR2_SNP55 | A/G | 0.0017 | 0.0896216 | 14.15 |
|  | Pto-PPR2_SNP60 | C/T | 9.29E-04 | 0.0599266 | 12.19 |
|  | Pto-PPR2_SNP64 | C/T | 0.001 | 0.0633266 | 15.14 |
|  | Pto-PPR2_SNP67 | C/T | 9.29E-04 | 0.0599266 | 12.19 |
|  | Pto-PPR2_SNP71 | A/G | 0.0013 | 0.0762782 | 14.69 |
