# Supplementary material for: Allelic Interactions among Pto-MIR475b and Its Four Target Genes Potentially Affect Growth and Wood Properties in Populus
Source: Front Plant Sci. 2017 Jun 21;8:1055. doi: 10.3389/fpls.2017.01055 (PMC5478899; doi:10.3389/fpls.2017.01055)
Supplement: Supplementary file 8 [file Table_6.DOCX]

**Table S6** Significant haplotypes from *Pto-MIR475b* and four targets associated with growth and wood properties in the association population

| **Gene** | **Blocks** | **Haplotypes** | **Phenotypes** | ***P_value*** | ***R^2^* (%)** |
| --- | --- | --- | --- | --- | --- |
| *Pto-MIR475b* |  |  |  |  |  |
|  | SNP1,2,3 | A-T-T | FW | 0.0631 | 18.11 |
|  | SNP1,2,3 | G-C-G | FW | 0.0336 | 18.11 |
|  | SNP8,9,10,11,12 | T-A-G-C-A | CC | 0.0684 | 1.46 |
|  | SNP8,9,10,11,12 | A-G-A-T-G | FW | 0.0808 | 8.52 |
|  | SNP8,9,10,11,12 | T-A-G-C-A | LC | 0.0615 | 1.37 |
|  | SNP17,18 | A-A | CC | 0.0934 | 0.38 |
|  | SNP17,18 | A-A | LC | 0.0815 | 1.69 |
|  | SNP17,18 | C-G | LC | 0.0990 | 1.69 |
| *Pto-PPR1* |  |  |  |  |  |
|  | SNP52,55,56 | C-A-A | FW | 0.0806 | 6.50 |
|  | SNP84,86 | T-T | LC | 0.0669 | 1.25 |
|  | SNP95,96,97,98,100 | T-G-G-C-T | LC | 0.0807 | 6.87 |
|  | SNP124,126 | T-T | FW | 0.0753 | 15.81 |
|  | SNP124,126 | T-T | HeC | 0.0868 | 15.51 |
|  | SNP128,130 | T-A | FL | 0.0862 | 2.24 |
|  | SNP135,136 | A-C | CC | 0.0859 | 4.26 |
|  | SNP135,136 | C-A | FW | 0.0863 | 11.16 |
|  | SNP153,155 | G-A | FW | 0.0837 | 9.55 |
| *Pto-PPR2* |  |  |  |  |  |
|  | SNP6,7,9,10,11 | A-A-A-A-A | FW | 0.0922 | 10.22 |
|  | SNP22,24,25,26,27 | G-G-A-A-C | CC | 0.0904 | 8.70 |
|  | SNP22,24,25,26,27 | A-T-T-G-T | FW | 0.0926 | 12.42 |
|  | SNP34,36,37,39,41 | A-T-C-C-G | CC | 0.0518 | 7.34 |
|  | SNP34,36,37,39,41 | T-A-T-A-A | FW | 0.0908 | 20.55 |
|  | SNP54,58,61 | A-G-C | FW | 0.0806 | 15.53 |
|  | SNP68,69,70,72 | G-C-T-C | FW | 0.0831 | 23.17 |
|  | SNP112,113 | G-A | H | 0.0916 | 0.47 |
|  | SNP112,113 | T-G | H | 0.0918 | 0.47 |
|  | SNP112,113 | G-A | LC | 0.0801 | 3.26 |
|  | SNP112,113 | T-G | LC | 0.0801 | 3.26 |
| *Pto-PPR3* |  |  |  |  |  |
|  | SNP9,11 | C-T | LC | 0.0936 | 6.80 |
|  | SNP9,11 | C-T | Hc | 0.0391 | 3.19 |
|  | SNP9,11 | C-T | CC | 0.0849 | 5.00 |
|  | SNP9,11 | T-A | HeC | 0.0705 | 4.28 |
|  | SNP9,11 | C-T | HeC | 0.0187 | 4.28 |
|  | SNP32,33 | C-C | LC | 0.0139 | 0.30 |
|  | SNP32,33 | C-C | HC | 0.0421 | 1.01 |
|  | SNP32,33 | C-C | CC | 0.0310 | 1.58 |
|  | SNP32,33 | C-C | HeC | 0.0426 | 1.06 |
|  | SNP40,41 | C-C | LC | 0.0602 | 0.56 |
|  | SNP40,41 | T-A | LC | 0.0602 | 0.56 |
|  | SNP46,47,48 | T-T-T | CC | 0.0637 | 10.36 |
|  | SNP50,51,52,53 | G-C-T-T | CC | 0.0807 | 4.42 |
|  | SNP54,55 | G-A | FW | 0.0995 | 0.15 |
|  | SNP56,57 | C-C | FW | 0.0956 | 1.55 |
|  | SNP56,57 | T-G | FW | 0.0956 | 1.55 |
| *Pto-PPR4* |  |  |  |  |  |
|  | SNP59,60,61,62 | C-G-T-T | FW | 0.0921 | 22.10 |
|  | SNP63,64,65 | T-C-G | FW | 0.0633 | 4.21 |
|  | SNP75,76,79,83,84 | C-A-C-T-C | CC | 0.0554 | 9.21 |
|  | SNP75,76,79,83,84 | T-T-A-C-T | FW | 0.0880 | 1.30 |
|  | SNP117,118,119,120 | A-G-A-C | FW | 0.0919 | 12.09 |
|  | SNP122,123,125 | T-G-T | FW | 0.0840 | 7.56 |
