# Supplementary material for: Allelic Interactions among Pto-MIR475b and Its Four Target Genes Potentially Affect Growth and Wood Properties in Populus
Source: Front Plant Sci. 2017 Jun 21;8:1055. doi: 10.3389/fpls.2017.01055 (PMC5478899; doi:10.3389/fpls.2017.01055)
Supplement: Supplementary file 9 [file Table_7.DOCX]

**Table S7** The SNP pairs and their main effects detected from *Pto-MIR475b* and the four targets under an epistasis model in the association population of *P. tomentosa.*

| **Traits** | **Attribute A** | **Attribute B** | **Effect of interaction (%)** | **Information gain (%)** |
| --- | --- | --- | --- | --- |
| CC (%) |  |  |  |  |
|  | Pto-PPR1_SNP85 | Pto-PPR1_SNP65 | 0.28 | -2.68 |
|  | Pto-PPR2_SNP74 | Pto-PPR1_SNP85 | 3.17 | 0.16 |
|  | Pto-PPR2_SNP74 | Pto-PPR1_SNP65 | 1.14 | 0.93 |
|  | Pto-PPR3_SNP28 | Pto-PPR1_SNP85 | 0.56 | -6.95 |
|  | Pto-PPR3_SNP28 | Pto-PPR1_SNP65 | 0.72 | -3.99 |
|  | Pto-PPR3_SNP28 | Pto-PPR2_SNP74 | 1.68 | -3.07 |
|  | Pto-PPR4_SNP126 | Pto-PPR3_SNP28 | 0.4 | -5.55 |
|  | Pto-PPR4_SNP126 | Pto-PPR4_SNP49 | 0.97 | -3.46 |
|  | Pto-PPR4_SNP126 | Pto-PPR1_SNP85 | 0.88 | -3.33 |
|  | Pto-PPR4_SNP126 | Pto-PPR2_SNP74 | 0.08 | -1.36 |
|  | Pto-PPR4_SNP126 | Pto-PPR1_SNP65 | 0.88 | -0.52 |
|  | Pto-PPR4_SNP49 | Pto-PPR3_SNP28 | 2.19 | -5.55 |
|  | Pto-PPR4_SNP49 | Pto-PPR1_SNP85 | 2.3 | -3.69 |
|  | Pto-PPR4_SNP49 | Pto-PPR2_SNP74 | 0 | -3.23 |
|  | Pto-PPR4_SNP49 | Pto-PPR1_SNP65 | 0.87 | -2.32 |
| DBH (cm) |  |  |  |  |
|  | Pto-PPR1_SNP115 | Pto-PPR1_SNP37 | 0.05 | -0.1 |
|  | Pto-PPR2_SNP114 | Pto-PPR1_SNP37 | 2.38 | -1.82 |
|  | Pto-PPR2_SNP114 | Pto-PPR1_SNP115 | 2.38 | -1.66 |
| FL (mm) |  |  |  |  |
|  | Pto-PPR4_SNP78 | Pto-PPR1_SNP118 | 4.38 | -4.1 |
|  | Pto-PPR2_SNP95 | Pto-PPR1_SNP118 | 4.38 | -3.98 |
|  | Pto-PPR4_SNP121 | Pto-PPR4_SNP18 | 3.14 | -3.55 |
|  | Pto-PPR4_SNP18 | Pto-PPR1_SNP118 | 9.81 | -2.47 |
|  | Pto-PPR4_SNP121 | Pto-PPR2_SNP95 | 0.42 | -2.35 |
|  | Pto-PPR4_SNP121 | Pto-PPR4_SNP78 | 0.56 | -2.32 |
|  | Pto-PPR4_SNP18 | Pto-PPR2_SNP92 | 2.16 | -2.15 |
|  | Pto-PPR4_SNP121 | Pto-PPR2_SNP92 | 0.77 | -2.08 |
|  | Pto-PPR4_SNP18 | Pto-PPR2_SNP95 | 2.3 | -1.94 |
|  | Pto-PPR2_SNP92 | Pto-PPR1_SNP118 | 7.14 | -1.29 |
|  | Pto-PPR4_SNP121 | Pto-PPR1_SNP118 | 9.7 | -1.11 |
|  | Pto-PPR4_SNP78 | Pto-PPR2_SNP92 | 0.23 | -0.27 |
|  | Pto-PPR4_SNP78 | Pto-PPR2_SNP95 | 0.28 | -0.15 |
|  | Pto-PPR2_SNP95 | Pto-PPR2_SNP92 | 2.15 | 1.76 |
|  | Pto-PPR4_SNP78 | Pto-PPR4_SNP18 | 6.8 | 2.45 |
| FW (µm) |  |  |  |  |
|  | Pto-PPR1_SNP115 | Pto-PPR1_SNP13 | 0.07 | -0.51 |
|  | Pto-PPR1_SNP115 | Pto-PPR1_SNP28 | 0.09 | -0.37 |
|  | Pto-PPR1_SNP28 | Pto-PPR1_SNP13 | 0.05 | -0.16 |
|  | Pto-PPR4_SNP108 | Pto-PPR1_SNP115 | 0 | -6.2 |
|  | Pto-PPR4_SNP108 | Pto-PPR1_SNP13 | 0.2 | -5.74 |
|  | Pto-PPR4_SNP108 | Pto-PPR1_SNP28 | 0.1 | -5.72 |
|  | Pto-PPR4_SNP108 | Pto-PPR4_SNP65 | 2.27 | -5.04 |
|  | Pto-PPR4_SNP126 | Pto-PPR4_SNP108 | 0.15 | -6.94 |
|  | Pto-PPR4_SNP126 | Pto-PPR4_SNP65 | 0.88 | -1.98 |
|  | Pto-PPR4_SNP126 | Pto-PPR1_SNP13 | 0.07 | -1.41 |
|  | Pto-PPR4_SNP126 | Pto-PPR1_SNP28 | 0.03 | -1.33 |
|  | Pto-PPR4_SNP126 | Pto-PPR1_SNP115 | 2.38 | 0.64 |
|  | Pto-PPR4_SNP65 | Pto-PPR1_SNP115 | 1.31 | -0.65 |
|  | Pto-PPR4_SNP65 | Pto-PPR1_SNP13 | 1.53 | -0.16 |
|  | Pto-PPR4_SNP65 | Pto-PPR1_SNP28 | 1.53 | -0.05 |
| H (m) |  |  |  |  |
|  | Pto-PPR1_SNP13 | Pto-MIR475b_SNP12 | 1.11 | -3.5 |
|  | Pto-PPR2_SNP33 | Pto-MIR475b_SNP12 | 2.42 | -4.04 |
|  | Pto-PPR2_SNP33 | Pto-PPR1_SNP13 | 0.88 | -1.29 |
|  | Pto-PPR3_SNP16 | Pto-MIR475b_SNP12 | 0.01 | -5.91 |
|  | Pto-PPR3_SNP16 | Pto-PPR1_SNP13 | 0.56 | -1.06 |
|  | Pto-PPR3_SNP16 | Pto-PPR2_SNP33 | 4.68 | 1.21 |
|  | Pto-PPR4_SNP35 | Pto-MIR475b_SNP12 | 2.03 | -2.85 |
|  | Pto-PPR4_SNP35 | Pto-PPR3_SNP16 | 0.08 | -1.81 |
|  | Pto-PPR4_SNP35 | Pto-PPR2_SNP33 | 0.85 | -1.59 |
|  | Pto-PPR4_SNP35 | Pto-PPR1_SNP13 | 0.55 | -0.04 |
|  | Pto-PPR4_SNP50 | Pto-MIR475b_SNP12 | 1.68 | -4.06 |
|  | Pto-PPR4_SNP50 | Pto-PPR3_SNP16 | 0.42 | -2.34 |
|  | Pto-PPR4_SNP50 | Pto-PPR1_SNP13 | 0 | -1.45 |
|  | Pto-PPR4_SNP50 | Pto-PPR2_SNP33 | 2.38 | -0.93 |
|  | Pto-PPR4_SNP50 | Pto-PPR4_SNP35 | 0.85 | -0.88 |
| HC (%) |  |  |  |  |
|  | Pto-PPR1_SNP97 | Pto-MIR475b_SNP5 | 0 | -4.07 |
|  | Pto-PPR3_SNP7 | Pto-MIR475b_SNP5 | 1.05 | -4.02 |
|  | Pto-PPR3_SNP7 | Pto-PPR1_SNP97 | 2.3 | -1.09 |
|  | Pto-PPR4_SNP108 | Pto-MIR475b_SNP5 | 0.54 | -8.08 |
|  | Pto-PPR4_SNP108 | Pto-PPR1_SNP97 | 0.15 | -6.78 |
|  | Pto-PPR4_SNP108 | Pto-PPR3_SNP7 | 4.45 | -3.48 |
| LC (%) |  |  |  |  |
|  | Pto-PPR1_SNP108 | Pto-PPR1_SNP74 | 0.88 | -1.12 |
|  | Pto-PPR1_SNP108 | Pto-PPR1_SNP61 | 1.99 | -0.38 |
|  | Pto-PPR1_SNP74 | Pto-PPR1_SNP61 | 1.89 | 0.51 |
|  | Pto-PPR2_SNP128 | Pto-PPR1_SNP108 | 0.09 | -3.9 |
|  | Pto-PPR2_SNP128 | Pto-PPR1_SNP61 | 0.23 | -3.15 |
|  | Pto-PPR2_SNP128 | Pto-PPR1_SNP74 | 0.28 | -2.73 |
|  | Pto-PPR3_SNP15 | Pto-PPR2_SNP128 | 1.15 | -7.69 |
|  | Pto-PPR3_SNP15 | Pto-PPR1_SNP61 | 0.68 | -6.54 |
|  | Pto-PPR3_SNP15 | Pto-PPR1_SNP74 | 4.08 | -2.76 |
|  | Pto-PPR3_SNP15 | Pto-PPR1_SNP108 | 6.83 | -1 |
|  | Pto-PPR3_SNP42 | Pto-PPR2_SNP128 | 0.08 | -5.25 |
|  | Pto-PPR3_SNP42 | Pto-PPR3_SNP15 | 4.84 | -4.33 |
|  | Pto-PPR3_SNP42 | Pto-PPR1_SNP74 | 1.31 | -2.03 |
|  | Pto-PPR3_SNP42 | Pto-PPR1_SNP108 | 2.77 | -1.56 |
|  | Pto-PPR3_SNP42 | Pto-PPR1_SNP61 | 2.3 | -1.41 |
| MFA (º) |  |  |  |  |
|  | Pto-PPR1_SNP101 | Pto-MIR475b_SNP2 | 2.38 | -2.59 |
|  | Pto-PPR1_SNP101 | Pto-PPR1_SNP48 | 6.1 | 3.71 |
|  | Pto-PPR1_SNP112 | Pto-MIR475b_SNP2 | 0.93 | -6.63 |
|  | Pto-PPR1_SNP112 | Pto-PPR1_SNP48 | 1.66 | -3.33 |
|  | Pto-PPR1_SNP112 | Pto-PPR1_SNP101 | 6.83 | -0.54 |
|  | Pto-PPR1_SNP125 | Pto-PPR1_SNP112 | 0.34 | -4.76 |
|  | Pto-PPR1_SNP125 | Pto-PPR1_SNP101 | 0.28 | -2.21 |
|  | Pto-PPR1_SNP125 | Pto-MIR475b_SNP2 | 1.42 | -1.27 |
|  | Pto-PPR1_SNP125 | Pto-PPR1_SNP48 | 1.14 | 1.02 |
|  | Pto-PPR1_SNP48 | Pto-MIR475b_SNP2 | 0.52 | -2.06 |
|  | Pto-PPR4_SNP121 | Pto-PPR1_SNP125 | 0.16 | -2.68 |
|  | Pto-PPR4_SNP121 | Pto-MIR475b_SNP2 | 3.6 | -1.71 |
|  | Pto-PPR4_SNP121 | Pto-PPR1_SNP48 | 1.26 | -1.48 |
|  | Pto-PPR4_SNP121 | Pto-PPR1_SNP112 | 6.41 | -1.31 |
|  | Pto-PPR4_SNP121 | Pto-PPR1_SNP101 | 4.84 | -0.28 |
| V (m^3^) |  |  |  |  |
|  | Pto-PPR1_SNP101 | Pto-PPR1_SNP37 | 2.3 | -0.2 |
|  | Pto-PPR2_SNP74 | Pto-PPR1_SNP101 | 2.3 | -0.13 |
|  | Pto-PPR2_SNP74 | Pto-PPR1_SNP37 | 0.33 | 0.01 |
|  | Pto-PPR2_SNP97 | Pto-PPR1_SNP101 | 2.3 | -0.13 |
|  | Pto-PPR2_SNP97 | Pto-PPR1_SNP37 | 0.33 | 0.01 |
|  | Pto-PPR2_SNP97 | Pto-PPR2_SNP74 | 1.18 | 0.93 |
|  | Pto-PPR2_SNP99 | Pto-PPR1_SNP101 | 0.33 | -2.39 |
|  | Pto-PPR2_SNP99 | Pto-PPR2_SNP74 | 0.42 | -0.13 |
|  | Pto-PPR2_SNP99 | Pto-PPR2_SNP97 | 0.42 | -0.13 |
|  | Pto-PPR2_SNP99 | Pto-PPR1_SNP37 | 0.68 | 0.06 |
